# The GSK3β/Mcl-1 axis is regulated by both FLT3-ITD and Axl and determines the apoptosis induction abilities of FLT3-ITD inhibitors

**DOI:** 10.1038/s41420-023-01317-0

**Published:** 2023-02-04

**Authors:** Yang Qiu, Ying Li, Meng Chai, Huiming Hua, Rui Wang, Samuel Waxman, Yongkui Jing

**Affiliations:** 1 Liaoning Key Lab of Targeting Drugs for Hematological Malignancies, Department of Pharmacology, Shenyang Pharmaceutical University, Shenyang, 110016 China; 2 School of Traditional Chinese Materia Medica, Shenyang Pharmaceutical University, Shenyang, 110016 China; 3 The Division of Hematology/Oncology, Department of Medicine, The Tisch Cancer Institute, Icahn School of Medicine at Mount Sinai, New York, NY 10029 USA

**Keywords:** Acute myeloid leukaemia, Pharmacodynamics

## Abstract

Acute myeloid leukemia (AML) patients with FLT3-ITD mutations are associated with poor prognosis. FLT3-ITD inhibitors are developed and result in transient disease remission, but generally resistance develops. We propose that resistance occurs due to apoptosis evasion. We compared the abilities of five clinically used FLT3-ITD inhibitors, namely, midostaurin, crenolanib, gilteritinib, quizartinib, and sorafenib, to induce apoptosis. These drugs inhibit FLT3-ITD and induce apoptosis. Apoptosis induction is associated with GSK3β activation, Mcl-1 downregulation, and Bim upregulation. Sorafenib-resistant MOLM-13/sor cells have the secondary D835Y mutation and increased Axl signaling pathway with cross-resistance to quizartinib. Gilteritinib and crenolanib inhibit both FLT3-ITD and Axl and induce apoptosis in MOLM-13/sor cells, in which they activate GSK3β and downregulate Mcl-1. Inactivation of GSK3β through phosphorylation and inhibitors blocks apoptosis and Mcl-1 reduction. The Axl/GSK3β/Mcl-1 axis works as a feedback mechanism to attenuate apoptosis of FLT3-ITD inhibition. Homoharringtonine decreases the protein levels of Mcl-1, FLT3-ITD, and Axl. Moreover, it synergistically induces apoptosis with gilteritinib in vitro and prolongs survival of MOLM-13/sor xenografts. The GSK3β/Mcl-1 axis works as the hub of FLT3-ITD inhibitors and plays a critical role in resistance against FLT3-ITD AML-targeted therapy.

## Introduction

Acute myeloid leukemia (AML) is a highly heterogeneous incurable disease that is classified based on molecular and genetic alterations [1]. Mutations in the FMS-like tyrosine kinase 3 (*FLT3*) gene are the most common mutations in AML [2]. Internal tandem duplication (ITD) mutations of *FLT3* occur in about 25% of AML cases and are associated with poor prognosis and increased risk of relapse [3]. Point mutations in the *FLT3* tyrosine kinase domain (TKD) occur in about 10% of AML cases, but are not associated with prognosis [4]. Both FLT3-ITD and FLT3-TKD are autophosphorylated and activated without FLT3 ligand. Although FLT3-ITD and FLT3-TKD activate the PI3K/AKT/mTOR and RAS/MEK/ERK signaling pathways [5], FLT3-ITD also activates the JAK/STAT5 signaling [6]. Therefore, FLT3-ITD and FLT3-TKD activate some different signaling pathways, which could account for their different impact on therapy prognosis. A large group of FLT3-ITD inhibitors have been developed and are used in the clinic [7]. Among these inhibitors, midostaurin was approved for newly diagnosed FLT3-ITD AML in combination with chemotherapy and gilteritinib was approved for resistant and relapsed FLT3-ITD AML [8, 9]. Sorafenib, an approved drug for kidney and liver cancer, is used off-label for FLT3-ITD AML patients [10]. Two more selective FLT3-ITD inhibitors, quizartinib and crenolanib, are actively being tested in phase III clinical trials [11, 12]. All of these inhibit FLT3-ITD with different abilities of inhibiting FLT3-TKD and they achieve transient disease remission with general resistance and disease relapse [13]. The therapeutic outcome needs to be improved by overcoming the resistance. It has been proposed that resistance to quizartinib and sorafenib develops because of secondary mutations of FLT3-ITD in the TKD [14]. However, gilteritinib and crenolanib inhibit both FLT3-ITD and FLT3-TKD with similar efficacies in FLT3-ITD AML patients as selective FLT3-ITD inhibitors [7]. Therefore, we believe that FLT3-ITD-independent signaling pathways overdrive the downstream signaling pathway(s) to mediate resistance and cause disease relapse.

Evasion of apoptosis is a primary cause of leukemogenesis and chemotherapy failure in AML [15]. The regulation of apoptosis is controlled by antiapoptotic and proapoptotic proteins [16]. Bax and Bak are essential effectors of apoptosis that are blocked by Bcl-2/Bcl-xL/Mcl-1. BH3-only proteins relay upstream apoptotic signals to initiate apoptosis by either activating Bax/Bak directly or inactivating Bcl-2/Bcl-xL/Mcl-1 [17]. In response to apoptotic signals, the BH3 activators (Bid, Bim, PUMA, and Noxa) directly activate Bax/Bak to induce Bax/Bak homo-oligomerization, leading to apoptosis [18]. FLT3-ITD upregulates transcription of *MCL1* and represses transcription of *BIM* and *PUMA* through unique pathways [19, 20]. The stabilities of these proteins are also regulated through phosphorylation by ERK and AKT, downstream signaling members of FLT3-ITD activation [21]. GSK3β is a main substrate of AKT and is inactivated by phosphorylation [22]. GSK3β has been reported to phosphorylate Mcl-1, PUMA, and Bax to regulate their stabilities and/or activities [23–25]. Phosphorylated GSK3β is considered to be a negative regulator of apoptosis and is associated with poor prognosis in AML therapy [26, 27]. It has been found that Mcl-1 is essential for the development and sustained growth of AML [28], and withdrawal of IL-3 leads to apoptosis through GSK3β-mediated Mcl-1 degradation in myeloid cells [29]. We hypothesize that GSK3β plays an essential role in apoptosis induction upon FLT3-ITD inhibition and that failure to activate GSK3β causes resistance.

In this study, we determined the phosphorylation level of GSK3β in FLT3-ITD AML cells treated with five clinically used FLT3-ITD inhibitors and analyzed its association with their apoptosis induction abilities. We studied the roles of GSK3β in apoptosis induction by inactivating it through FLT3-ITD-independent pathways and explored the downstream members of the apoptosis induction pathway. We established a sorafenib-resistant cell line and dissected the resistance mechanisms by next-generation RNA sequencing (RNA-Seq) and tested the cross-resistance of the five FLT3-ITD inhibitors and investigated the alternative signaling pathways to inactivate GSK3β and block apoptosis.

## Results

### FLT3-ITD inhibitors induce apoptosis associated with inhibition of ERK and activation of GSK3β

**Fig. 1:**
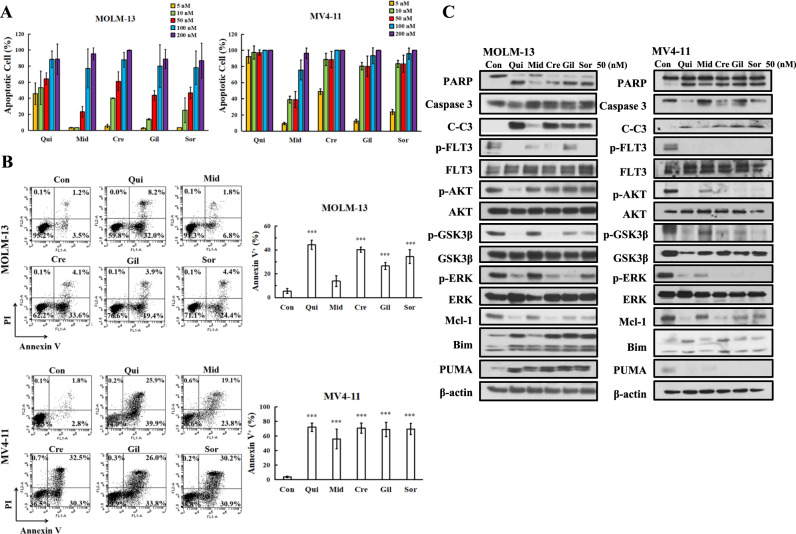
FLT3-ITD inhibitors induce apoptosis with reduced levels of Mcl-1 and p-GSK3β. **A** Apoptosis detected by AO-EB staining. **B** Apoptosis detected by Annexin V/PI staining. **C** Protein levels measured by Western blot analysis. MOLM-13 and MV4-11 cells were treated with each inhibitor at the indicated concentrations for 24 h. Column figures show the mean plus SD of three independent experiments. ***P < 0.001 compared with the control group.

Midostaurin, crenolanib, and gilteritinib are type I FLT3-ITD inhibitors, while quizartinib and sorafenib are type II FLT3-ITD inhibitors [30]. Midostaurin and sorafenib are multiple kinase inhibitors with inhibition of FLT3-ITD, PDGFR, VEGFR, and KIT. Quizartinib, crenolanib and gilteritinib are relative selective inhibitors of FLT3-ITD. Quizartinib and crenolanib inhibit PDGFR while gilteritinib inhibits Axl. Midostaurin, crenolanib, and gilteritinib inhibit FLT3-TKD [31]. All five inhibitors inhibited FLT3-ITD activity and cell growth at concentrations below 10 nM [32]. We analyzed apoptosis induction in MOLM-13 and MV4-11 cells based on morphological changes after AO/EB staining in a concentration range of 5–200 nM for each inhibitor. Both cell lines are sensitive to quizartinib at 5–10 nM. Quizartinib at 5 nM induced apoptosis in 45.73% of MOLM-13 cells and in 83.00% of MV4-11 cells. MV4-11 cells are more sensitive than MOLM-13 cells to the apoptosis-inducing effects of these inhibitors (Fig. 1A). The apoptosis induction abilities at 50 nM varied among these inhibitors and there is no difference at 100 nM. We selected 50 nM for further study and confirmed the apoptosis-inducing effects of each inhibitor at 50 nM in both MOLM-13 and MV4-11 cells by Annexin V/PI staining. Quizartinib, crenolanib, gilteritinib, and sorafenib exhibited a similar apoptosis induction ability at this concentration, and midostaurin was less effective (Fig. 1B).

The downstream ERK and AKT/GSK3β signaling pathways of FLT3-ITD were compared in both cell lines treated with 50 nM of each inhibitor. All five inhibitors decreased the levels of p-FLT3-ITD in both cell lines. Quizartinib, sorafenib, crenolanib, and gilteritinib decreased the levels of p-ERK, p-AKT, and p-GSK3β in both cell lines (Fig. 1C). Midostaurin had a weak effect on the levels of these phosphorylated proteins in MOLM-13 cells. The cleaved PARP and caspase-3 levels were correlated with upregulation of Bim and downregulation of Mcl-1 in both lines. MV4-11 cells were more sensitive than MOLM-13 cells to apoptosis induction with less induction of Bim and without induction of PUMA. At 10 nM only quizartinib induces apoptosis in MOML-13 cells which is associated with downregulation of p-GSK3β and Mcl-1 proteins (Fig. S1).

### TPA and GSK3β inhibitors block FLT3-ITD inhibitor-induced apoptosis

**Fig. 2:**
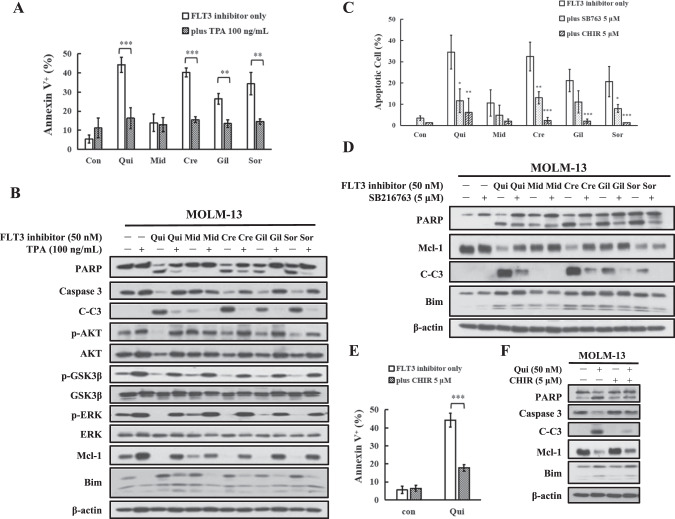
Inactivation of GSK3β blocks apoptosis induction of FLT3-ITD inhibitors. **A** MOLM-13 cells were pretreated with 100 ng/mL TPA and then cotreated with each inhibitor at 50 nM for 24 h. Apoptotic cells were examined by Annexin V/PI staining. **B** Western blot analysis of protein changes in TPA-pretreated MOLM-13 cells followed by each inhibitor. **C** MOLM-13 cells pretreated with GSK3β inhibitor CHIR-99021 and SB216763 for 4 h and then with FLT3-ITD inhibitors for 24 h. Apoptotic cells were detected by AO-EB staining. **D** Protein regulation of MOLM-13 cells treated with SB216763 and FLT3-ITD inhibitors for 24 h. **E** MOLM-13 cells were treated with 5 μM CHIR-99021 and quizartinib for 24 h. Apoptotic cells were detected by Annexin V/PI staining. **F** Protein regulation of MOLM-13 cells treated with CHIR-99021 and quizartinib for 24 h. *P < 0.05, **P < 0.01, ***P < 0.001 compared with the FLT3-ITD inhibitor group.

TPA has been shown to activate p-ERK and to increase the levels of Mcl-1 in leukemia cells [33, 34]. Pretreatment with TPA decreased the apoptosis induction ability of quizartinib, sorafenib, crenolanib, and gilteritinib (Fig. 2A). Midostaurin has minimal apoptosis induction ability at this concentration, which was not influenced by TPA pretreatment. The levels of p-AKT, p-GSK3β, p-ERK, and Mcl-1 were increased while the levels of Bim were decreased in TPA-pretreated MOLM-13 cells. The decrease in p-AKT, p-GSK3β, p-ERK, and Mcl-1 expression and the increase in Bim expression by these inhibitors were blocked by TPA pretreatment, but the upregulation of PUMA was not blocked. The inhibition of PARP and caspase-3 cleavage was correlated with p-GSK3β and Mcl-1 restoration as well as Bim repression (Fig. 2B).

We then tested the role of GSK3β in the apoptosis induction using two GSK3β inhibitors, SB216763 and CHIR-99021, which significantly attenuated apoptosis induction by sorafenib, crenolanib, gilteritinib, and quizartinib at 50 nM in both MOLM-13 and MV4-11 cells based on the AO/EB assay (Fig. 2C). SB216763 blocked the cleavage of PARP and caspase-3 and reversed Mcl-1 downregulation, but not Bim induction (Fig. 2D). Because SB216763 interferes with Annexin V staining for the apoptosis assay, we used CHIR-99021 to confirm the inhibition of quizartinib-induced apoptosis using Annexin V staining (Fig. 2E). CHIR-99021 reduced the apoptosis induction ability of quizartinib and reversed the Mcl-1 downregulation, but not Bim induction (Fig. 2F). To test the role of Bim in apoptosis induction, *BIM* siRNA was used. Silencing Bim did not attenuate quizartinib-induced apoptosis with Mcl-1 downregulation (Fig. S2A, B). These data suggest that GSK3β-mediated Mcl-1 downregulation plays an essential role in the apoptosis induction of FLT3-ITD inhibitors.

### Sorafenib-resistant MOLM-13/sor cells have increased protein levels of FLT3 and Axl signaling pathways

**Fig. 3:**
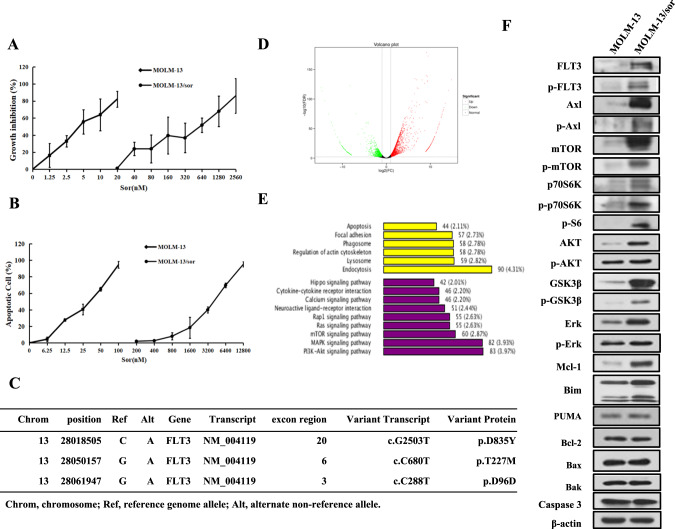
Differentially expressed genes and proteins of MOLM-13 and MOLM-13/sor cells. **A** Growth inhibition rate of MOLM-13 and MOLM-13/sor cells treated with sorafenib for 72 h. **B** Apoptosis of MOLM-13 and MOLM-13/sor cells treated with sorafenib for 24 h as detected by AO-EB staining. **C** WES analysis showing FLT3 mutations in MOLM-13/sor cells. **D** Volcano plot of the differentially expressed genes in MOLM-13/sor cells compared to MOLM-13 cells. The green and red dots represent upregulated and downregulated genes, respectively. **E** KEGG classification of differentially expressed genes. The ordinate shows the KEGG pathway and the abscissa shows the number of genes under this pathway and its proportion in the total number of genes. **F** Differentially expressed proteins analyzed by Western blot analysis in MOLM-13 and MOLM-13/sor cells.

The sorafenib-resistant subclone MOLM-13/sor was established by continuous stimulation with sorafenib. The GI_50_ of sorafenib in MOLM-13 cells was 5.03 nM and that of MOLM-13/sor cells was 435.18 nM, with a drug resistance factor of 86.51 (Fig. 3A). Sorafenib induced apoptosis starting at 12.5 nM with maximal apoptosis induction of 94.33% at 100 nM in MOLM-13, but it did not induce apoptosis in MOLM-13/sor until the concentration was increased to 800 nM (Fig. 3B). Since it is difficult to colonize this line we used the pool. WES analysis revealed three new mutations of the FLT3-ITD gene, including the reported D835Y point mutation and two unreported T227M and D96D point mutations, in MOLM-13/sor cells (Fig. 3C). RNA-Seq analysis revealed 4490 differentially expressed genes between MOLM-13 and MOLM-13/sor cells, with 2946 genes being upregulated and 1544 genes being downregulated in MOLM-13/sor cells. A volcano plot of the relative expression levels of differentially expressed genes in MOLM-13/sor cells compared with MOLM-13 cells is shown in Fig. 3D. KEGG pathway analysis of differentially expressed genes revealed that 5 of the top 15 most significantly enriched KEGG pathways were associated with apoptosis (2.11%), PI3K/AKT signaling (3.97%), MAPK signaling (3.93%), mTOR signaling (2.63%), and RAS signaling (2.63%) (Fig. 3E). We found that the protein levels of mTOR, p70S6K, FLT3, AKT, GSK3β, ERK, Mcl-1, and Bim were increased. The levels of p-mTOR, p-p70S6K, p-S6, p-AKT, p-ERK, and p-GSK3β were also increased. Axl is a membrane tyrosine kinase isolated from chronic myeloid leukemia cells and alternatively activates RAS/ERK and AKT/GSK3β signaling [35]. Axl has been found to be activated in sorafenib-resistant hepatocellular carcinoma [36]. We compared the levels of Axl and p-Axl and found that both were increased in MOLM-13/sor cells compared to MOLM-13 cells (Fig. 3F). These results suggest that activated FLT3-ITD signaling, Axl signaling, and protein translation contribute to sorafenib resistance.

### The Axl/GSK3β signaling axis causes resistance to quizartinib, but not to crenolanib and gilteritinib

**Fig. 4:**
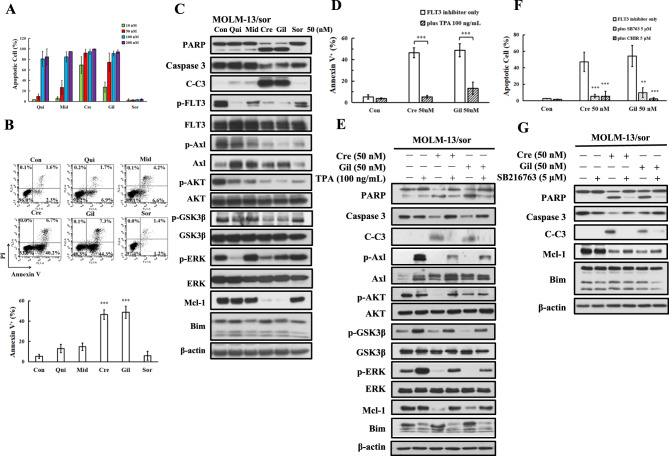
Apoptosis induction and protein regulation of FLT3-ITD inhibitors in MOLM-13/sor cells. **A** Apoptosis detected by AO-EB staining in MOLM-13/sor cells treated with each inhibitor for 24 h. **B** Apoptosis detected by Annexin V/PI staining in MOLM-13/sor cells treated by 50 nM of each inhibitor. **C** Protein regulation in MOLM-13/sor cells treated with each inhibitor at 50 nM for 24 h. **D** MOLM-13/sor cells pretreated with 100 ng/mL TPA and then with crenolanib and gilteritinib at 50 nM for 24 h. Apoptosis was detected by Annexin V/PI staining. **E** Apoptosis-related proteins analyzed by Western blot analysis. **F** MOLM-13/sor cells were pretreated with 5 μM GSK3β inhibitor CHIR-99021 or SB216763 for 4 h and then cotreated with crenolanib and gilteritinib for 24 h. Apoptotic cells were detected by AO-EB staining. **G** Protein regulation in MOLM-13/sor cells treated with SB216763 in combination with crenolanib and gilteritinib for 24 h. *P < 0.05, **P < 0.01, ***P < 0.001 compared with the FLT3 inhibitor group.

We compared the apoptosis induction abilities of quizartinib, midostaurin, crenolanib, and gilteritinib in MOLM-13/sor cells (Fig. 4A). MOLM-13/sor cells became insensitive to lower concentrations of quizartinib, but they had increased sensitivity to crenolanib and gilteritinib. MOLM-13/sor cells were equally sensitive to quizartinib, midostaurin, crenolanib, and gilteritinib at 100 nM (Fig. 4A). At a concentration of 50 nM, only crenolanib and gilteritinib induced apoptosis in MOLM-13/sor cells based on Annexin V staining (Fig. 4B). Quizartinib downregulated p-FLT3-ITD, p-AKT, and p-ERK, but not p-Axl, without cleavage of PARP and caspase-3. Crenolanib and gilteritinib downregulated p-FLT3-ITD, p-AKT, p-ERK, and p-Axl and induced cleavage of PARP and caspase-3 (Fig. 4C). The apoptosis induction ability of crenolanib and gilteritinib is negatively correlated with p-Axl, p-GSK3β, and Mcl-1 levels (Fig. 4C).

TPA was also shown to activate Axl in AML cells [35]. We analyzed the effects of TPA on the Axl, ERK, and AKT signaling pathways in both MOLM-13 and MOLM-13/sor cell lines, and we found that TPA increased the protein levels of Axl. TPA also increased the levels of p-Axl, p-ERK, p-AKT, and p-GSK3β in both cell lines. TPA increased the levels of Mcl-1 in MOLM-13 cells, but not in MOLM-13/sor cells, probably due to the higher basal levels of Mcl-1 in MOLM-13/sor cells (Fig. S3). TPA reduced crenolanib-induced apoptosis from 46.4% to 5.3% and gilteritinib-induced apoptosis from 48.8% to 13.2% (Fig. 4D). TPA reversed the downregulation of Mcl-1 and p-GSK3β by crenolanib and gilteritinib treatment (Fig. 4E). We also noted that TPA repressed Bim expression in the combination-treated cells. The GSK3β inhibitors SB216763 and CHIR-99021 blocked crenolanib- and gilteritinib-induced apoptosis (Fig. 4F). SB216763 blocked Mcl-1 downregulation and PARP cleavage. The levels of Bim were not influenced by crenolanib and gilteritinib alone or in combination with SB216763 (Fig. 4G). These data further suggest that GSK3β-mediated Mcl-1 downregulation causes apoptosis in crenolanib- and gilteritinib-treated MOLM-13/sor cells.

IL-3 withdrawal has been found to activate GSK3β and decrease Mcl-1 levels to induce apoptosis in IL-3-dependent 32D myeloid cells [29]. Transfection of FLT3-ITD and FLT3-TKD into 32D cells stimulates cell growth without the need for IL-3. We took advantage of FLT3-ITD- and FLT3-TKD-transfected 32D/FLT3-ITD and 32D/FLT3-TKD cells to test the role of IL-3 in regulating apoptosis [37]. Both 32D/FLT3-ITD and 32D/FLT3-TKD cells growing without IL-3 are sensitive to crenolanib- and gilteritinib-induced apoptosis, which were blocked by addition of IL-3 (Fig. S4A, C). IL-3 blocked the downregulation of Mcl-1 and p-GSK3β upon crenolanib and gilteritinib treatment in 32D/FLT3-TKD cells (Fig. S4B). These data indicate that FLT3 inhibitor-induced apoptosis can be blocked by cytokines through inactivating GSK3β in different ways.

### The Axl inhibitor BGB324 enhances quizartinib-induced apoptosis in MOLM-13/sor cells

BGB324 is an Axl inhibitor that is currently being investigated in clinical trials [38]. We tested if MOLM-13 and MOLM-13/sor cells are sensitive to BGB324 apoptosis induction. BGB324 at 0.5 μM induced apoptosis in MOLM-13/sor cells, but not in MOLM-13 cells (Fig. S5A). BGB324 at 2 μM induced cleavage of PARP and caspase-3 and reduced p-AKT, p-ERK, p-GSK3β, and Mcl-1 levels in MOLM-13/sor cells (Fig. S5B).

We then tested if BGB324 can be used to overcome quizartinib resistance in MOLM-13/sor cells. We selected 0.125 μM BGB324, which reduces p-Axl levels, and 50 nM quizartinib, which does not induce apoptosis in MOLM-13/sor cells (Fig. S5C). BGB324 plus quizartinib induced apoptosis by exerting a synergistic effect with enhanced Mcl-1 downregulation (Fig. S5D). We noticed increased protein levels of Axl in BEG324- and quizartinib-treated cells. These data suggest a feedback mechanism of Axl is activated by these inhibitors. Silencing of *AXL* using siRNA has a profound effect, enhancing both quizartinib-induced apoptosis and the downregulation of Mcl-1 (Fig. S5E). To decrease the levels of p-GSK3β, silencing of *AXL* is more effective than BEG324 treatment. We then silenced *MCL1* using siRNA and found that *MCL1* silencing induced apoptosis in MOLM-13/sor cells, which could not be enhanced by quizartinib (Fig. S6A), suggesting that Mcl-1 reduction is essential to induce apoptosis in MOLM-13/sor cells. We then compared the effects of *MCL1* silencing on apoptosis in MOLM-13, MV4-11, THP-1, and K562 cells. *MCL1* silencing induced apoptosis in MOLM-13 and MV4-11 cells, but not in THP-1 and K562 cells (Fig. S6B). These data suggest that FLT3-ITD AML cells rely on Mcl-1 to survive, in contrast to other types of AML cells.

### Homoharringtonine represses FLT3-ITD, Axl, and Mcl-1 protein levels and enhances crenolanib- and gilteritinib-induced apoptosis

**Fig. 5:**
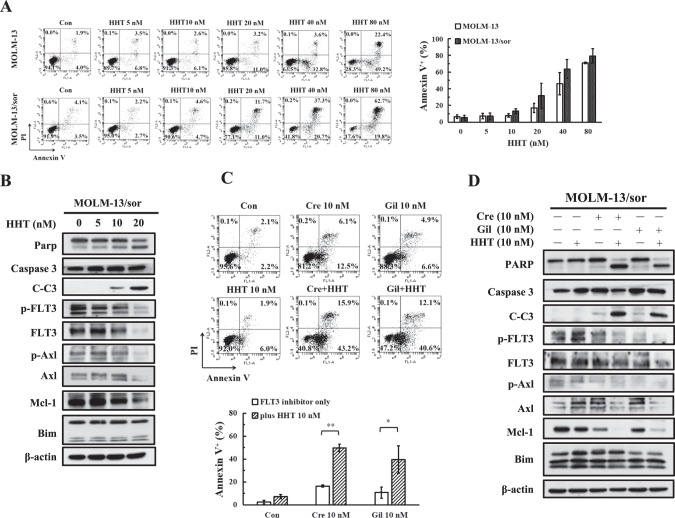
Apoptosis induction of HHT in combination with crenolanib and gilteritinib in MOLM-13/sor cells. **A** Apoptosis of MOLM-13 and MOLM-13/sor cells treated with HHT at the indicated concentrations for 24 h and detected by Annexin V/PI staining. **B** Protein regulation of HHT in MOLM-13/sor cells. **C** Apoptosis of MOLM-13/sor cells treated with 10 nM HHT plus 10 nM crenolanib or gilteritinib for 24 h detected by Annexin V/PI staining. *P < 0.05, **P < 0.01 compared with the FLT3 inhibitor group. **D** Protein regulation of MOLM-13/sor cells treated by HHT plus crenolanib or gilteritinib.

Although crenolanib and gilteritinib induced apoptosis in MOLM-13/sor cells, they also increased the protein levels of Axl. Also, this line has increased levels of a group of proteins that could attenuate the apoptosis induction (Fig. 3E). HHT is a protein translation inhibitor and induces apoptosis in AML cells with Mcl-1 downregulation, and it is approved for treatment of chronic myeloid leukemia resistant to BCR-ABL inhibitors [39, 40]. It has been reported that FLT3-ITD cells are sensitive to HHT [41]. We analyzed the ability of HHT to induce apoptosis in MOLM-13 and MOLM-13/sor cells. HHT at 5–80 nM induced apoptosis in both cell lines in a dose-dependent manner. Both MOLM-13 and MOLM-13/sor cells were sensitive to HHT-induced apoptosis without significant difference (Fig. 5A). We focused on MOLM-13/sor cells to test the combination of HHT with crenolanib and gilteritinib. HHT at 10-20 nM induced PARP cleavage in MOLM-13/sor cells and decreased the protein levels of FLT3-ITD, Axl, and Mcl-1 without affecting the levels of Bim (Fig. 5B). HHT at 10 nM in combination with 10 nM crenolanib or gilteritinib induced apoptosis in about 40–50% of cells, while each alone only induced apoptosis in less than 10% of cells (Fig. 5C). HHT in combination with crenolanib or gilteritinib augmented cleavage of PARP and caspase-3 and downregulated Mcl-1 and Axl at the protein level (Fig. 5D).

### HHT and gilteritinib have enhanced antileukemia effects in MOLM-13/sor xenografts

**Fig. 6:**
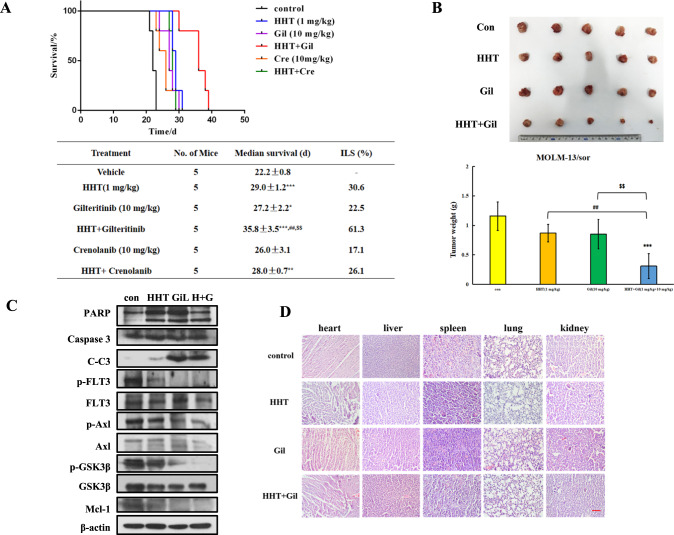
The combined effects of HHT with gilteritinib in MOLM-13/sor xenografts. **A** Survival curves of MOLM-13/sor xenografted mice inoculated through the tail vein and treated with HHT, gilteritinib, crenolanib, or their combination for 12 days. **B** Tumor image and tumor weights of MOLM-13/sor cells inoculated subcutaneously into NOD-SCID mice treated with HHT, gilteritinib, or the combination for 6 days. ***P < 0.001 compared with the control group. ^##^*P* < 0.01 compared with the HHT group. ^$$^*P* < 0.01 compared with the gilteritinib group. **C** Protein analysis in tumor tissue. **D** H&E staining of heart, liver, spleen, lung, and kidney of mice. The scale bar is 100 μM.

MOLM-13/sor cells inoculated into NOD-SCID were used to test the combined effects of HHT with gilteritinib and crenolanib in vivo. Mice inoculated with MOLM-13/sor cells through the tail vein were treated with HHT (1 mg/kg), gilteritinib (10 mg/kg), crenolanib (10 mg/kg), or HHT in combination with gilteritinib or crenolanib (Fig. 6A). The average survival time of the control group was 22.2 days. Both HHT and gilteritinib alone were effective, with ILS values of 30.6% and 22.5%, respectively. The combination of HHT and gilteritinib increased the ILS to 61.3% (Fig. 6A). Crenolanib alone is not effective and the combination with HHT did not show improvement at the same treatment regimen (Fig. 6A).

In order to test the protein regulation of HHT in combination with gilteritinib in vivo, MOLM-13/sor cells were inoculated subcutaneously and treated with HHT (1 mg/kg), gilteritinib (1 mg/kg), or their combination for six consecutive days. The tumor growth inhibition rates of HHT and gilteritinib alone were 24.9% and 26.5%, respectively, and that of HHT in combination with gilteritinib was 73.1% (Fig. 6B). HHT and gilteritinib alone as well as combined induced cleavage of PARP and caspase-3 with downregulation of Axl, FLT3-ITD, and Mcl-1 proteins (Fig. 6C). The H&E staining results of mouse tissue showed that this combination did not result in morphological toxicity in the heart, liver, spleen, lung, and kidney (Fig. 6D).

## Discussion

**Fig. 7:**
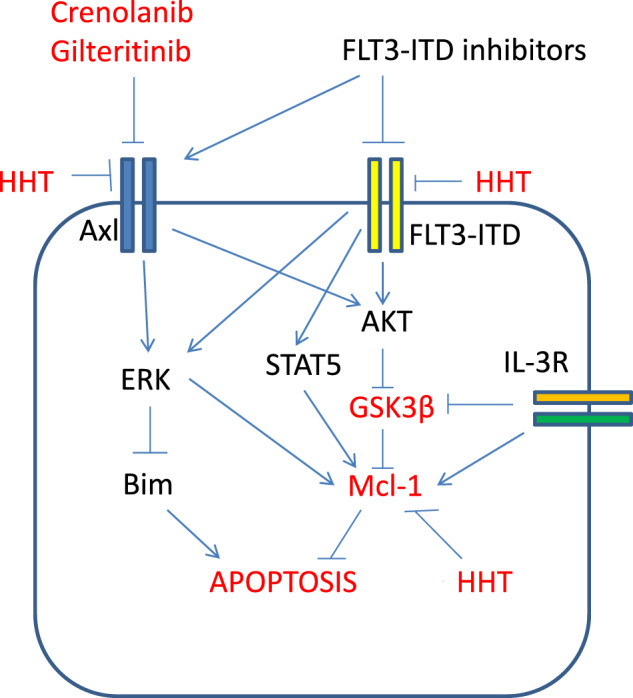
Mechanistic cascade of apoptosis induction by FLT3-ITD inhibitors and homoharringtonine (HHT). FLT3-ITD, Axl and IL-3 receptor inactivate GSK3b and increase Mcl-1 protein to block apoptosis. FLT3-ITD inhibitors activate GSK3b by inhibiting FLT3-ITD and Axl and then decrease Mcl-1 protein level. HHT decreases the protein levels of Mcl-1, FLT3-ITD and Axl, and enhances FLT3-ITD inhibitor-induced apoptosis.

We identified that the GSK3β/Mcl-1 axis plays an essential role in FLT3-ITD inhibitor-induced apoptosis and resistance, and it is regulated by multiple factors including FLT3-ITD, AXL and IL-3 (Fig. 7).

Mcl-1 is a short-life protein and plays an important role in regulating survival of neutrophils [42]. Mcl-1 levels are increased in AML cells and Mcl-1 plays a more important role than other Bcl-2 antiapoptotic proteins to maintain AML survival [28]. Mcl-1 direct inhibitors have not been successfully developed as therapeutics probably due to the unselective inhibition of leukemia and normal cells, and heart toxicity [43]. Selectively targeting Mcl-1 in AML cells is required. The stability of Mcl-1 is regulated by the ERK and AKT signaling pathways, which are activated in AML cells. ERK phosphorylates Mcl-1, increasing its stability [44], while GSK3β phosphorylates Mcl-1, decreasing its stability [29]. We found that FLT3-ITD inhibitor-induced apoptosis is associated with reduced GSK3β phosphorylation and Mcl-1 downregulation. Inactivation of GSK3β by TPA-induced phosphorylation and GSK3β inhibitors prevented Mcl-1 downregulation and blocked apoptosis. Since these inhibitors do not induce apoptosis in other types of AML cells, targeting FLT3-ITD could selectively induce apoptosis in FLT3-ITD AML cells through GSK3β activation-mediated Mcl-1 downregulation. We found that FLT3-ITD AML is dependent on Mcl-1 to survive. Directly silencing Mcl-1 induces apoptosis in MOLM-13 and MV4-11 cells, but not in THP-1 and K562 cells (Fig. S6). Bim has been shown to play a more important role than PUMA in FLT3-ITD inhibitor-mediated apoptosis [45]. We found that Bim is upregulated in MOLM-13 and MV4-11 cells (Fig. 1C). Although silencing Bim does not attenuate quizartinib-induced apoptosis (Fig. S2), Bim is an important partner of Mcl-1 to induce apoptosis, and Bim levels remain high in resistant cells. In the present study we did not elucidate the role of Bim in the apoptosis induction of FLT3-ITD inhibitors.

High levels of phosphorylated GSK3β are associated with poor prognosis in leukemia cells [26]. GSK3β could be inactivated through phosphorylation by multiple factors in myeloid cells [23]. Axl, a member of the TAM family (which includes TYRO3 and MER), plays an important role in the regulation of cellular processes such as proliferation, motility, and survival [46]. Overexpression of Axl is associated with poor prognosis and the development of resistance [47]. Axl has cooperative effects with FLT3-ITD in leukemogenesis and stimulates overlapping downstream signaling pathways [12, 48]. Sorafenib-resistant cells have increased levels of Axl and p-Axl as well as the secondary D835Y mutation (Fig. 3). This line is resistant to quizartinib, which can be overcome by treatment with Axl inhibitor and Axl siRNA through reducing Mcl-1 expression (Fig. S5). This line remains sensitive to gilteritinib and crenolanib, which both reduce p-Axl and p-FLT3-ITD, activate GSK3β, and downregulate Mcl-1. Therefore, dual Axl and FLT3-ITD inhibitors have an advantage in targeting FLT3-ITD selective inhibitor-resistant AML cells. In support of this, there is a report showing that sorafenib-resistant FLT3-ITD patients respond to gilteritinib [49]. However, gilteritinib still exhibits transient effects in FLT3-ITD AML patients. Although gilteritinib reduces p-Axl levels, we noticed that Axl protein expression is induced by all FLT3-ITD inhibitors. The upregulated Axl protein might serve as a feedback signal to attenuate the effects of these inhibitors. We found that Axl inhibitor also increases the protein levels of Axl and has a limited effect enhancing the apoptosis induction abilities of these inhibitors. It remains unclear how Axl protein is upregulated by these agents. We and other researchers have found activated mTOR translational signaling in resistant cell lines, which may lead to upregulation of Axl at the protein level [50, 51]. Furthermore, we found that TPA further upregulated Axl and p-GSK3β, and blocked the apoptosis induction ability of gilteritinib and crenolanib in sorafenib-resistant cells. The upregulated Axl may inactivate GSK3β and attenuate the ability of FLT3-ITD inhibitors.

HHT is a protein translation inhibitor and used for AML and CML treatment [39]. We and other group have found that HHT down-regulated Mcl-1 protein and induced apoptosis in AML HL-60 cell line [40, 52]. It has been found that FLT3-ITD cells are sensitive to HHT and have augmented effect with sorafenib [41]. Here we found that sorafenib-resistant cells were sensitive as parental cells to HHT-induced apoptosis with decreased protein levels of Axl, FLT3-ITD, and Mcl-1 (Fig. 5). HHT in combination with gilteritinib induce synergistic apoptosis and significantly prolong the sorafenib-resistant cell growth in xenografts. Due to the dual inhibition of protein and enzymatic activity of both FLT3-ITD and Axl by gilteritinib and HHT, this combination may have an advantage to be used for FLT3-ITD AML relapsed from other inhibitor treatment.

IL-3 withdrawal in 32D cells induces GSK3β-mediated Mcl-1 downregulation and apoptosis [29]. 32D cells transfected with FLT3-ITD and FLT3-TKD support cell growth without the need for IL-3 [37]. Gilteritinib and crenolanib induce apoptosis in both 32D/FLT3-ITD and 32D/FLT3-TKD cells, which is blocked by the addition of IL-3 (Fig. S4). Our data support that FLT3-ITD shares an overlapping signaling with IL-3. IL-3, IL-5, and GM-CSF share a common receptor beta chain and mediate the oncogenic activity of FLT3-ITD [53]. Hematopoietic cytokines IL-3 and GM-CSF have been found to mediate resistance to FLT3-ITD-targeted therapy [54]. These cytokines inactivate GSK3β through PKC-mediated phosphorylation and block Mcl-1 degradation [55]. Therefore, inactivation of GSK3β by cytokines might contribute to the resistance to dual FLT3-ITD and Axl inhibitors, and that antagonists of cytokines may be useful to improve the therapy of gilteritinib and/or to overcome its resistance.

## Materials and methods

### Reagents

Midostaurin, crenolanib, gilteritinib, quizartinib, sorafenib, CHIR-99021, and SB216763 were obtained from Selleck Chemicals (Houston, TX, USA). Homoharringtonine (HHT) injection solution was purchased from Minsheng Pharmaceutical Group (Hangzhou, China). Acridine orange (AO), 12-*O*-tetradecanoylphorbol 13-acetate (TPA), and ethidium bromide (EB) were obtained from Sigma-Aldrich (St. Louis, MO, USA). Antibodies against PARP and caspase-3 were purchased from BD Biosciences (Maryland, USA); antibodies against Mcl-1 (S-19), Bcl-2 (C-2), and β-actin (C-2) were acquired from Santa Cruz Biotechnology (San Diego, CA); antibodies against FLT3 (8F2), phospho-FLT3 (Tyr589/591)(30D4), Axl (C89E7), phospho-Axl (Tyr698), phospho-AKT (Ser473), phospho-GSK3β (Ser9), phospho-ERK (Thr202/Tyr204), PUMA, Bim (C34C5), and cleaved caspase-3 were obtained from Cell Signaling Technology, Inc. (Beverly, MA); and siRNAs for *AXL* (sc-29769), *MCL1* (sc-35877) and *BIM* (sc-29802) were obtained from Santa Cruz Biotechnology (San Diego, CA).

### Cell lines

MOLM-13 cells (with FLT3-ITD mutation) were obtained from DSMZ-Deutsche Sammlung von Mikroorganismen und Zellkulturen GmbH. MV4-11 (with FLT3-ITD mutation), THP-1 (MLL-AF9 translocation), and K562 (with BCR-ABL translocation) cells were obtained from the American Type Culture Collection. These cell lines were cultured in RPMI 1640 medium supplemented with 100 units/mL penicillin, 100 μg/mL streptomycin, 1 mM L-glutamine, and 10% (v/v) heat-inactivated FBS. 32D/FLT3-ITD and 32D/FLT3-TKD cells transfected with FLT3-ITD and FLT3-TKD, respectively, were provided by Dr. Naoe (Nagoya University Graduate School of Medicine) [37]. 32D/FLT3-ITD and 32D/FLT3-TKD cells were cultured in RPMI 1640 medium without IL-3. MOLM-13/sor resistant cells were established by continuous stimulation of MOLM-13 cells with sorafenib and maintained with 200 nM sorafenib; they were cultured for one week without sorafenib before the experiments.

### Apoptosis assays, Western blot analysis, and siRNA interference experiments

Apoptosis assays, Western blot analysis, and siRNA interference experiments were conducted as previously described [56, 57].

### Whole-exome sequencing and RNA-Seq analysis

Whole-exome sequencing (WES) and RNA-Seq were performed on MOLM-13 and MOLM-13/sor cells by Biomarker Technologies (Beijing, China). The DNA libraries were generated using the NimbleGen SeqCap EZ Human Exome V3 (Roche, Basel, Swiss). RNA was extracted from MOLM-13 and MOLM-13/sor cells according to the instruction manual of the TRIzol Reagent kit (Life technologies, California, USA). The analyses were performed as reported [58].

### In vivo treatment studies

Animal experiments were approved by the Animal Care and Use Committee of Shenyang Pharmaceutical University. The protocol was done as previously reported [57]. In the first experiment, 30 NOD-SCID mice (obtained from Beijing Weitonglihua, 8 weeks, male) were intraperitoneally injected with 150 mg/kg cyclophosphamide for two consecutive days, and each mouse was inoculated with 5 × 10^6^ MOLM-13/sor cells through the tail vein. After 3 days, mice were randomly divided into 6 groups (*n* = 5 mice per group) and treated with saline (control), HHT (1 mg/kg), gilteritinib (10 mg/kg), HHT plus gilteritinib, crenolanib (10 mg/kg), or HHT plus crenolanib. HHT was administered intraperitoneally once a day for 12 consecutive days. Gilteritinib and crenolanib were administered orally once a day for 12 consecutive days. The weight and survival time of mice were recorded, and the increase of life span (ILS) was calculated.

In a second experiment, 20 NOD-SCID mice were inoculated with 5 × 10^6^ MOLM-13/sor cells subcutaneously into the lateral armpits. When the tumor volume reached 100 mm^3^, mice were randomly divided into 4 groups (*n* = 5 mice per group) and treated with saline, HHT (1 mg/kg), gilteritinib (10 mg/kg), or HHT plus gilteritinib. HHT was administered intraperitoneally once a day, and gilteritinib was administered orally once a day for six consecutive days. Tumor size was measured every two days and tumors were dissected on day 7. Tumor tissues were used for Western blot analysis. The heart, liver, spleen, lung, and kidney were dissected and fixed in paraformaldehyde for 48 h, paraffin-embedded, sliced, and stained with an H&E staining kit using a standard protocol.

### Statistical analysis

GraphPad Prism software was used for statistical analysis. The Student *t*-test was used to determine the significance of differences between two groups. One-way ANOVA was used for multiple groups. *P* < 0.05 was considered to be statistically significant. The data are presented as mean ± SD.

## Supplementary information

Supplementary Figure Legends

Supplementary Figures

Original Data File

## Data Availability

All data needed to support the conclusions are included in this published article and supplementary materials.
