# Supplementary material for: The GSK3β/Mcl-1 axis is regulated by both FLT3-ITD and Axl and determines the apoptosis induction abilities of FLT3-ITD inhibitors
Source: Cell Death Discov. 2023 Feb 4;9:44. doi: 10.1038/s41420-023-01317-0 (PMC9899255; doi:10.1038/s41420-023-01317-0)
Supplement: Supplementary file 1 — Supplementary Figure Legends [file 41420_2023_1317_MOESM1_ESM.pdf]

Supplementary Figure legends

Figure S1. Apoptosis induction of FLT3 inhibitors at 10 nM in MOLM-13 cells. **A** Apoptosis detected by Annexin V/PI staining. **B** Protein levels measured by Western blot analysis. MOLM-13 cells were treated with each FLT3 inhibitor at 10 nM for 24 h. $^{**}P < 0.01$ compared with the control group.

Figure S2. Silencing *BIM* does not block quizartinib-induced apoptosis. MOLM-13 cells were silenced with *BIM* siRNA and treated with quizartinib at 50 nM for 24 h. The apoptotic cells were detected by Annexin V/PI staining (**A**) and protein was detected by Western blot analysis (**B**).

Figure S3. TPA stimulates multiple signaling pathways. MOLM-13 and MOLM-13/sor cells were treated with TPA for 24 h and the protein levels were detected by Western blot analysis.

Figure S4. IL-3 blocks crenolanib- and gilteritinib-induced apoptosis in 32D/FLT3-ITD and 32D/FLT3-TKD cells. 32D/FLT3-ITD and 32D/FLT3-TKD cells were pretreated with or without 2 ng/mL IL-3 for 4 h and then with crenolanib and gilteritinib for another 24 h. Apoptosis was detected by Annexin V/PI staining (**A**, **C**) and protein was analyzed by Western blot (**B**).

Figure S5. Axl inhibition overcomes quizartinib resistance. **A** MOLM-13 and MOLM-13/sor cells were treated with the Axl inhibitor BGB324 for 24 h. Apoptosis was detected by Annexin V/PI staining. **B**, Protein regulation measured by Western blot of MOLM-13/sor cells treated with BGB324 for 24 h. **C** MOLM-13/sor cells were treated with 0.125 μM BGB324 and 10 nM quizartinib for 24 h. Apoptosis detected by Annexin V/PI staining. $^{**}P < 0.01$ compared with the FLT3 inhibitor group. **D** Protein regulation of MOLM-13/sor cells treated with BGB324 and quizartinib for 24 h. **E** Axl was silenced with *AXL* siRNA in MOLM-12/sor cells that were subsequently treated with quizartinib. PARP cleavage was detected by Western blot.

Figure S6. Silencing *MCL1* induces apoptosis in MOLM-13, MOLM-13/sor, and MV4-11 cells. **A** MOLM-13/sor cells were silenced with *MCL1* siRNA and treated with 50 nM quizartinib. **B** MOLM-13, MV4-11, THP-1, and K562 cells were silenced with *MCL1* siRNA. Apoptotic cells were detected with Annexin V/PI staining and the protein levels were detected by Western blot analysis.
