# Supplementary figures and images for: The GSK3β/Mcl-1 axis is regulated by both FLT3-ITD and Axl and determines the apoptosis induction abilities of FLT3-ITD inhibitors
Source: Cell Death Discov. 2023 Feb 4;9:44. doi: 10.1038/s41420-023-01317-0 (PMC9899255; doi:10.1038/s41420-023-01317-0)

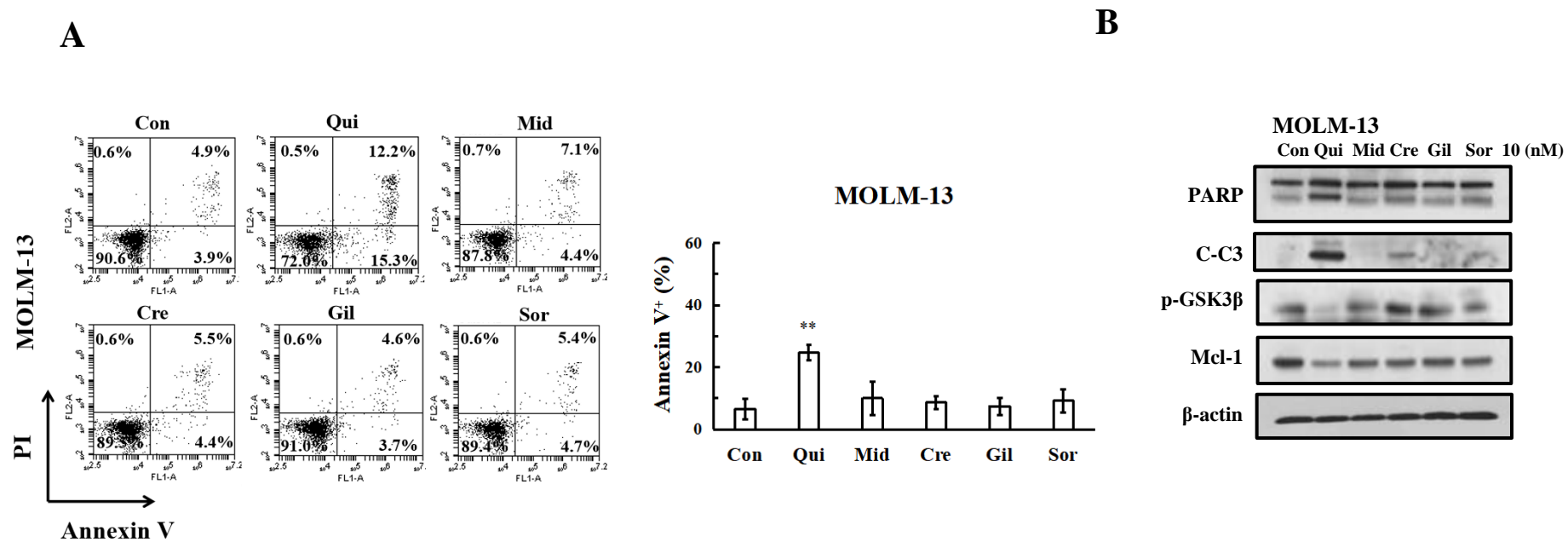

**Fig.S1**

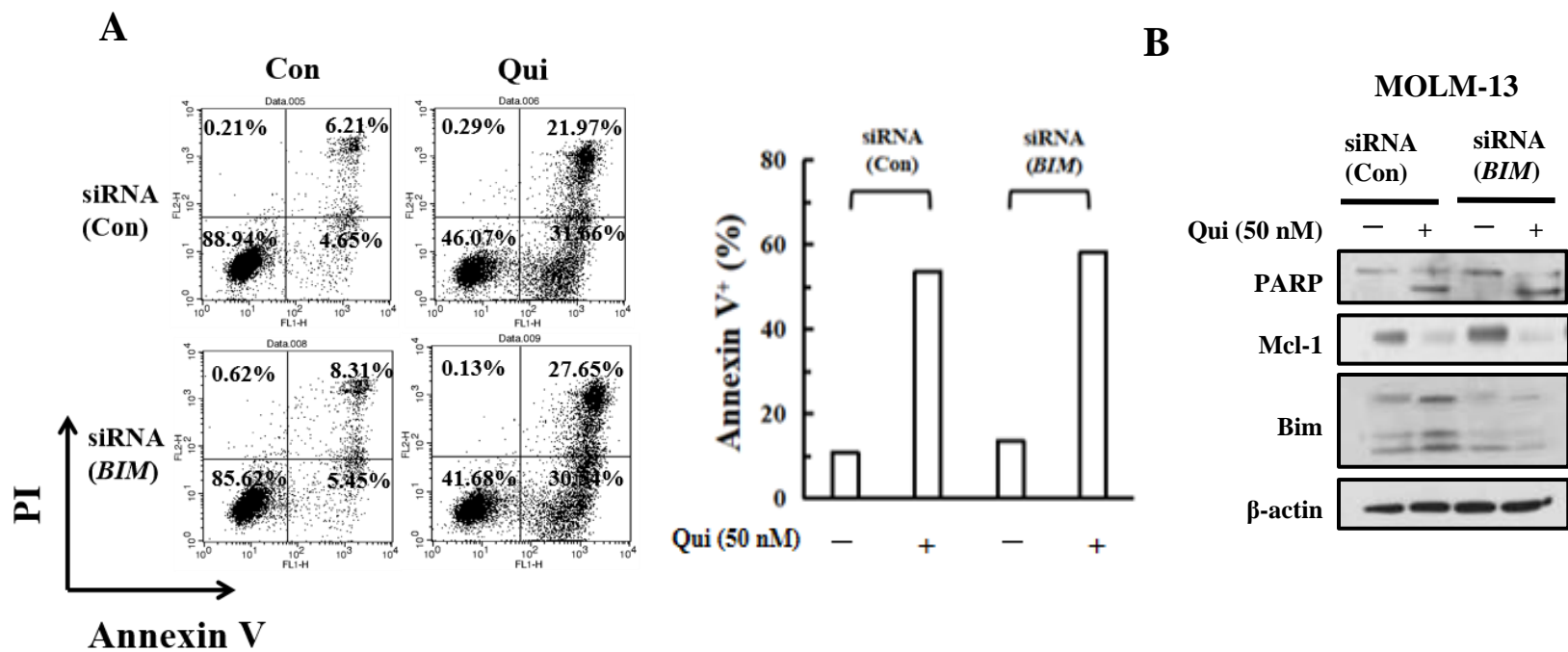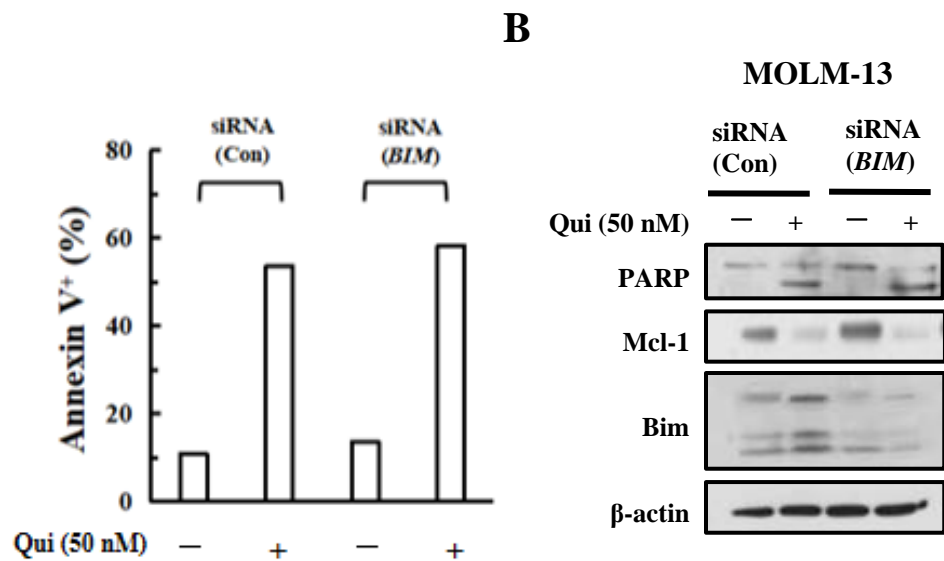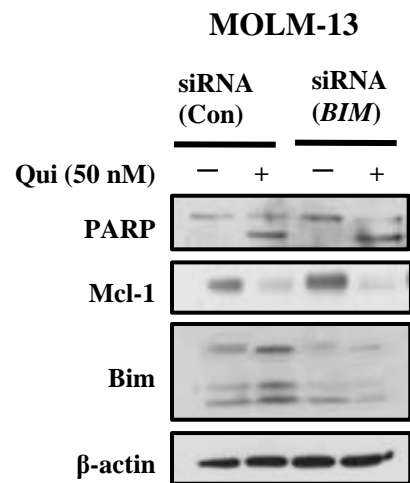

**Fig.S2**

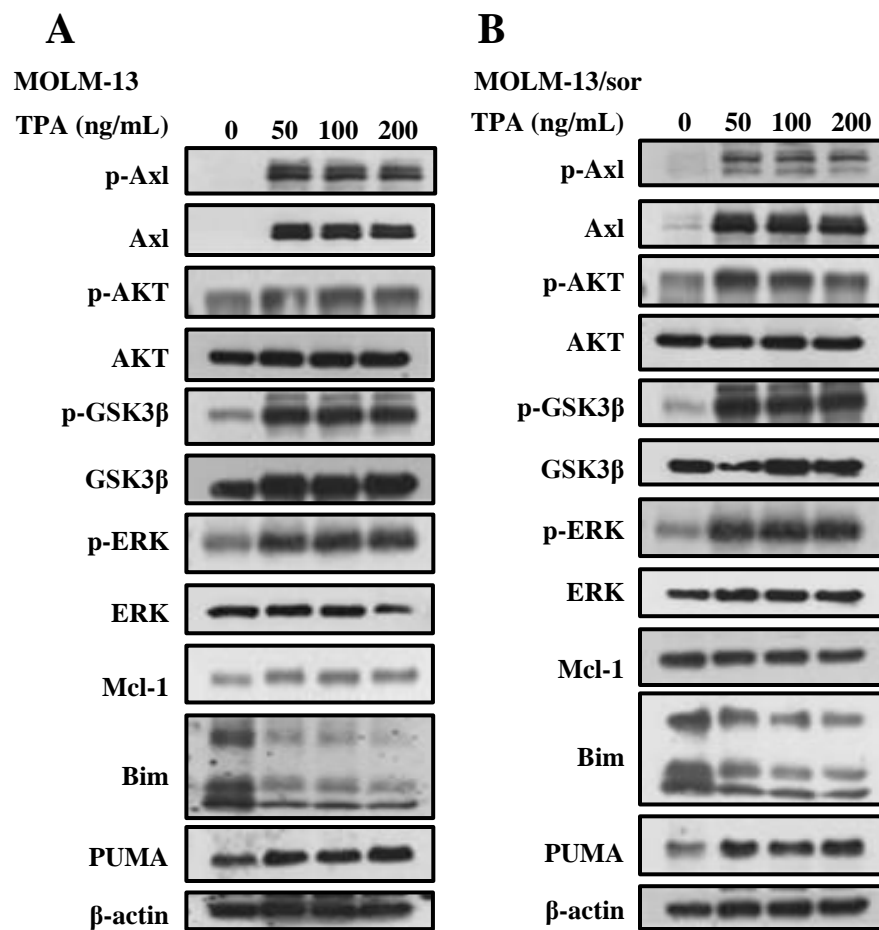

**Fig. S3**

**A**

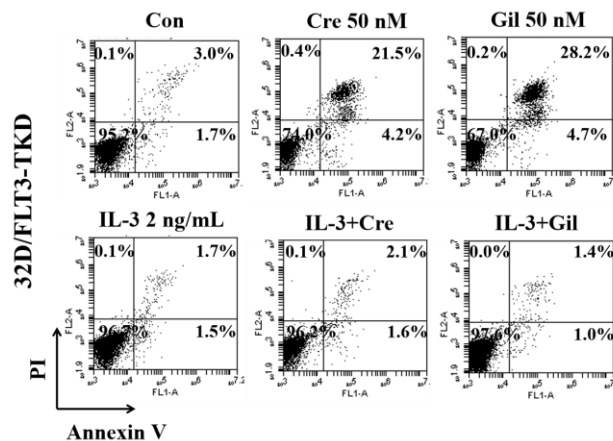

**B**

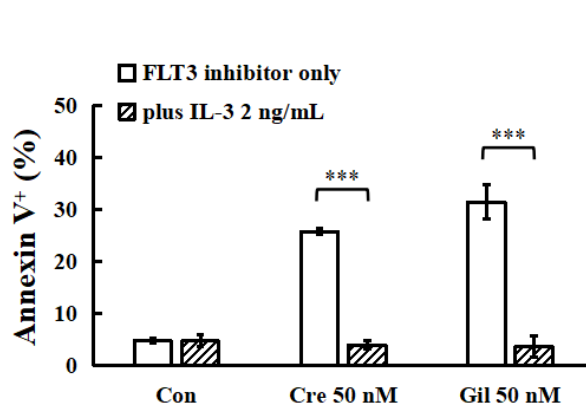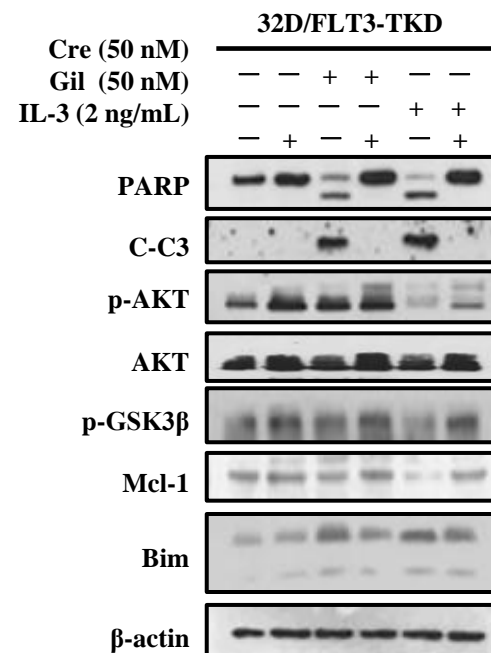

**C**

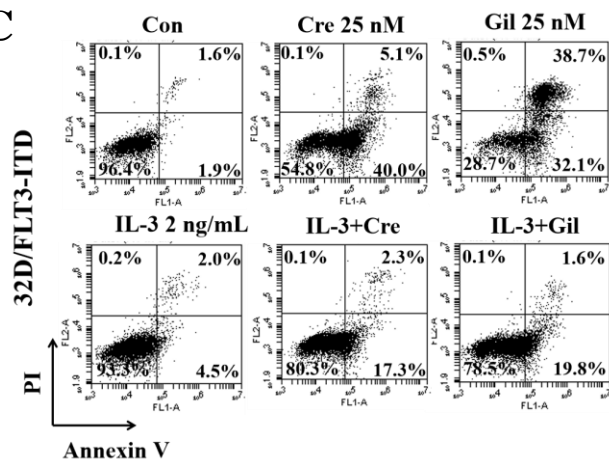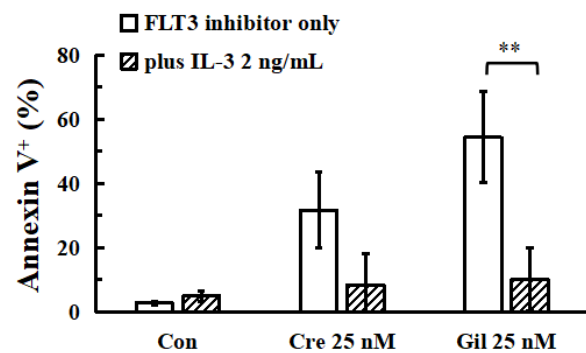

**Fig.S4**

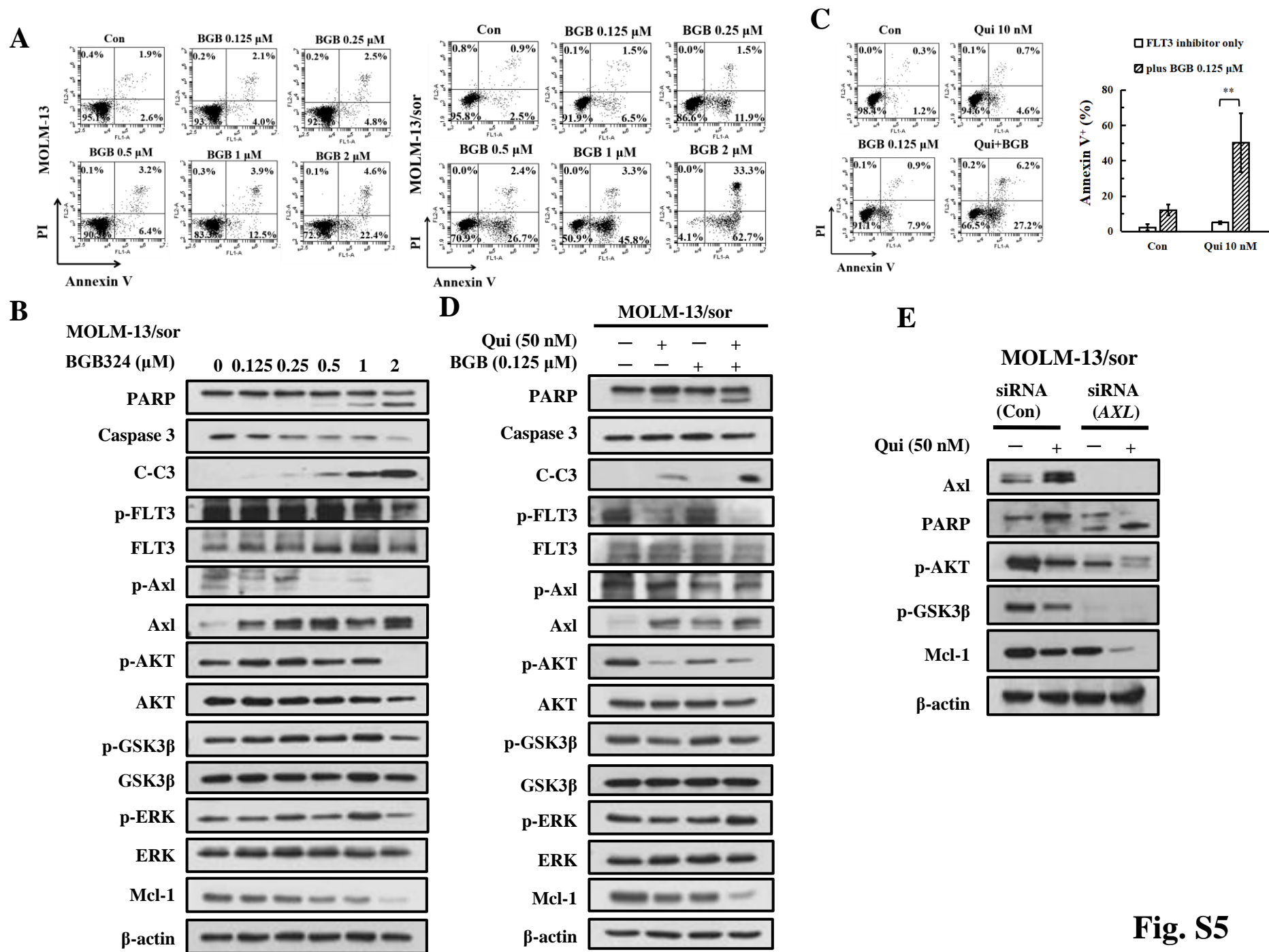

**Fig. S5**

**A**

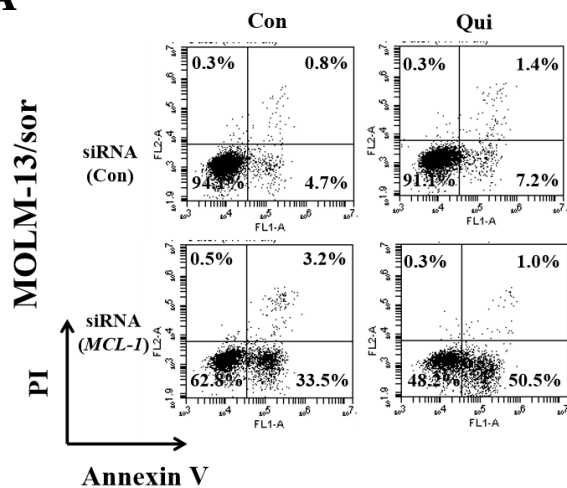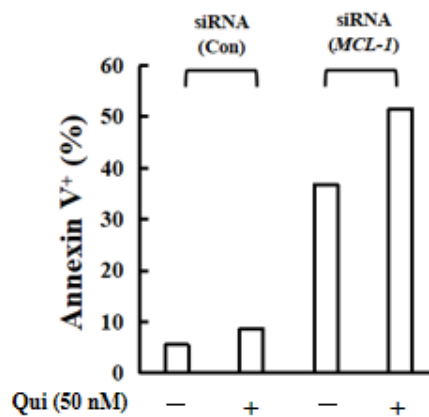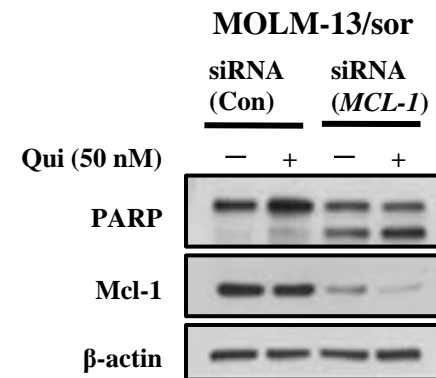

**B**

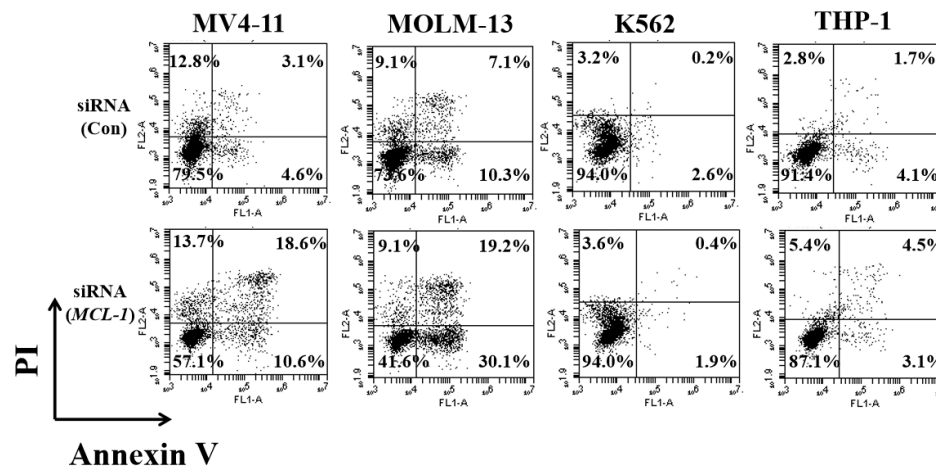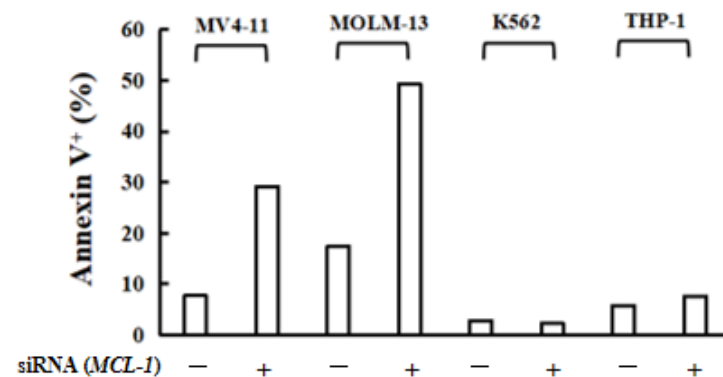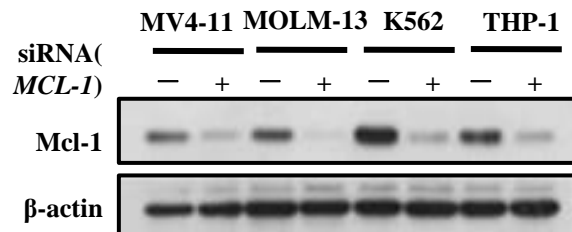

**Fig. S6**

Supplement: Supplementary file 2 — Supplementary Figures [file 41420_2023_1317_MOESM2_ESM.pdf]
