# Supplementary material for: The GSK3β/Mcl-1 axis is regulated by both FLT3-ITD and Axl and determines the apoptosis induction abilities of FLT3-ITD inhibitors
Source: Cell Death Discov. 2023 Feb 4;9:44. doi: 10.1038/s41420-023-01317-0 (PMC9899255; doi:10.1038/s41420-023-01317-0)

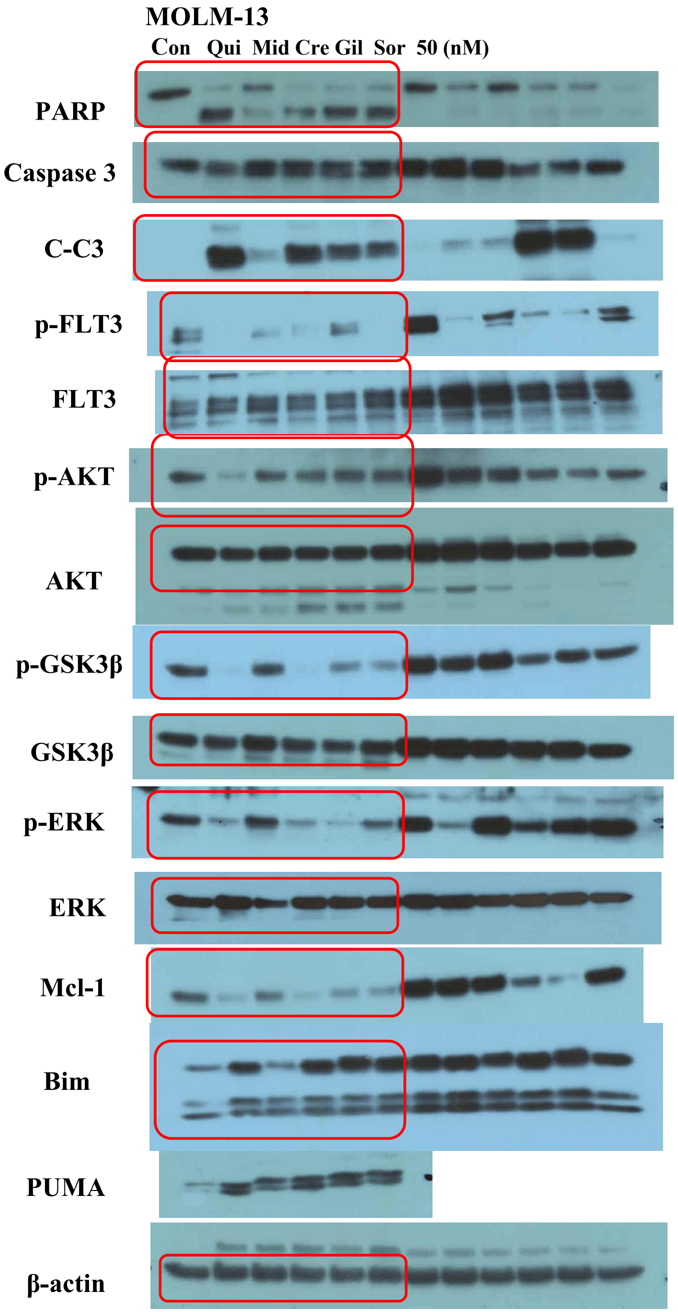
MOLM-13
Con Qui Mid Cre Gil Sor 50 (nM)
PARP
Caspase 3
C-C3
p-FLT3
FLT3
p-AKT
AKT
p-GSK3β
GSK3β
p-ERK
ERK
Mcl-1
Bim
PUMA
β-actin

Fig.1C

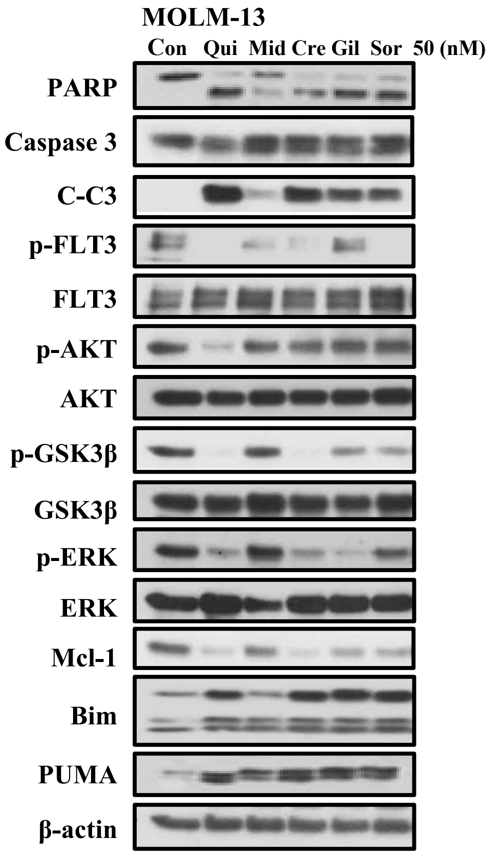
MOLM-13
Con Qui Mid Cre Gil Sor 50 (nM)
PARP
Caspase 3
C-C3
p-FLT3
FLT3
p-AKT
AKT
p-GSK3β
GSK3β
p-ERK
ERK
Mcl-1
Bim
PUMA
β-actin

Fig.1C

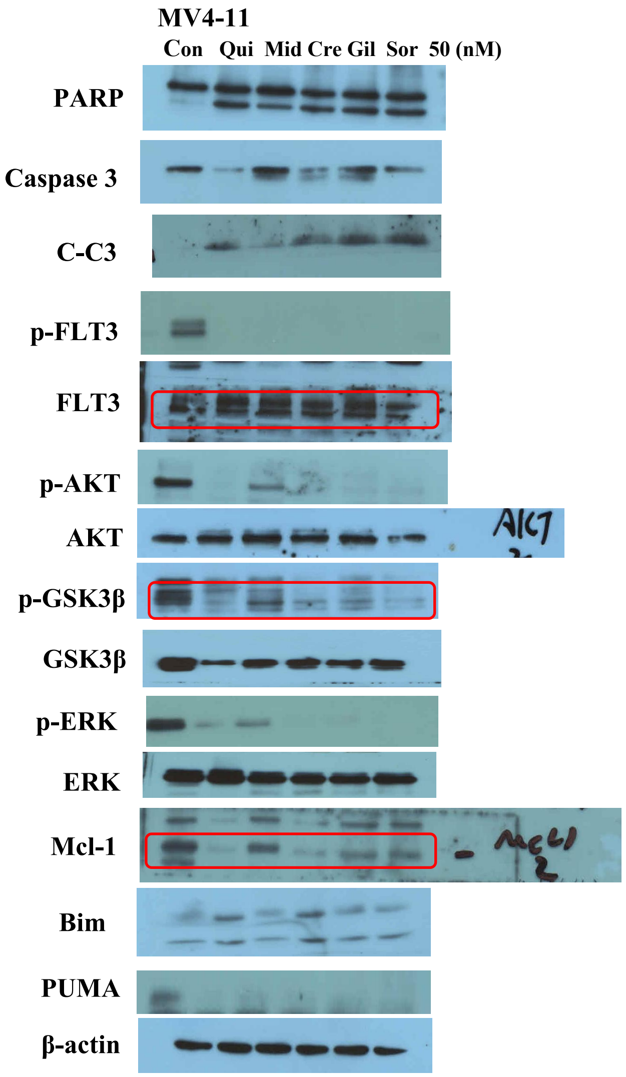

MV4-11
Con Qui Mid Cre Gil Sor 50 (nM)
PARP
Caspase 3
C-C3
p-FLT3
FLT3
p-AKT
AKT
p-GSK3β
GSK3β
p-ERK
ERK
Mcl-1
Bim
PUMA
β-actin

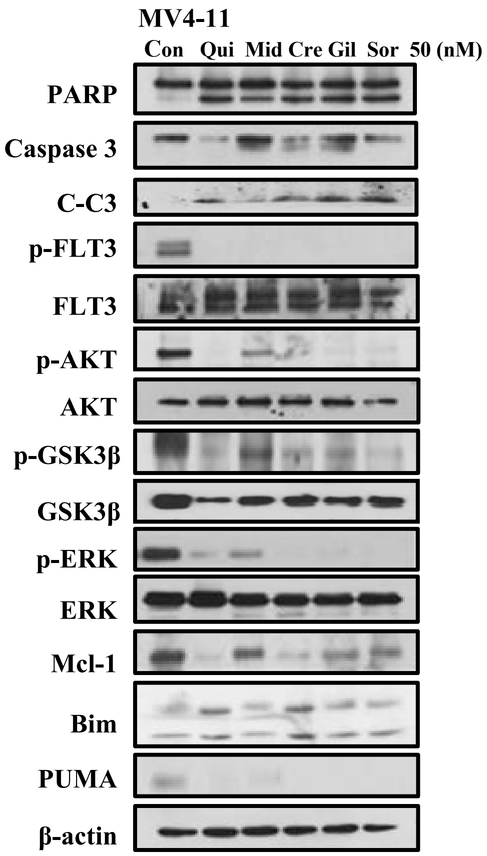

MV4-11
Con Qui Mid Cre Gil Sor 50 (nM)
PARP
Caspase 3
C-C3
p-FLT3
FLT3
p-AKT
AKT
p-GSK3β
GSK3β
p-ERK
ERK
Mcl-1
Bim
PUMA
β-actin

Fig.2B

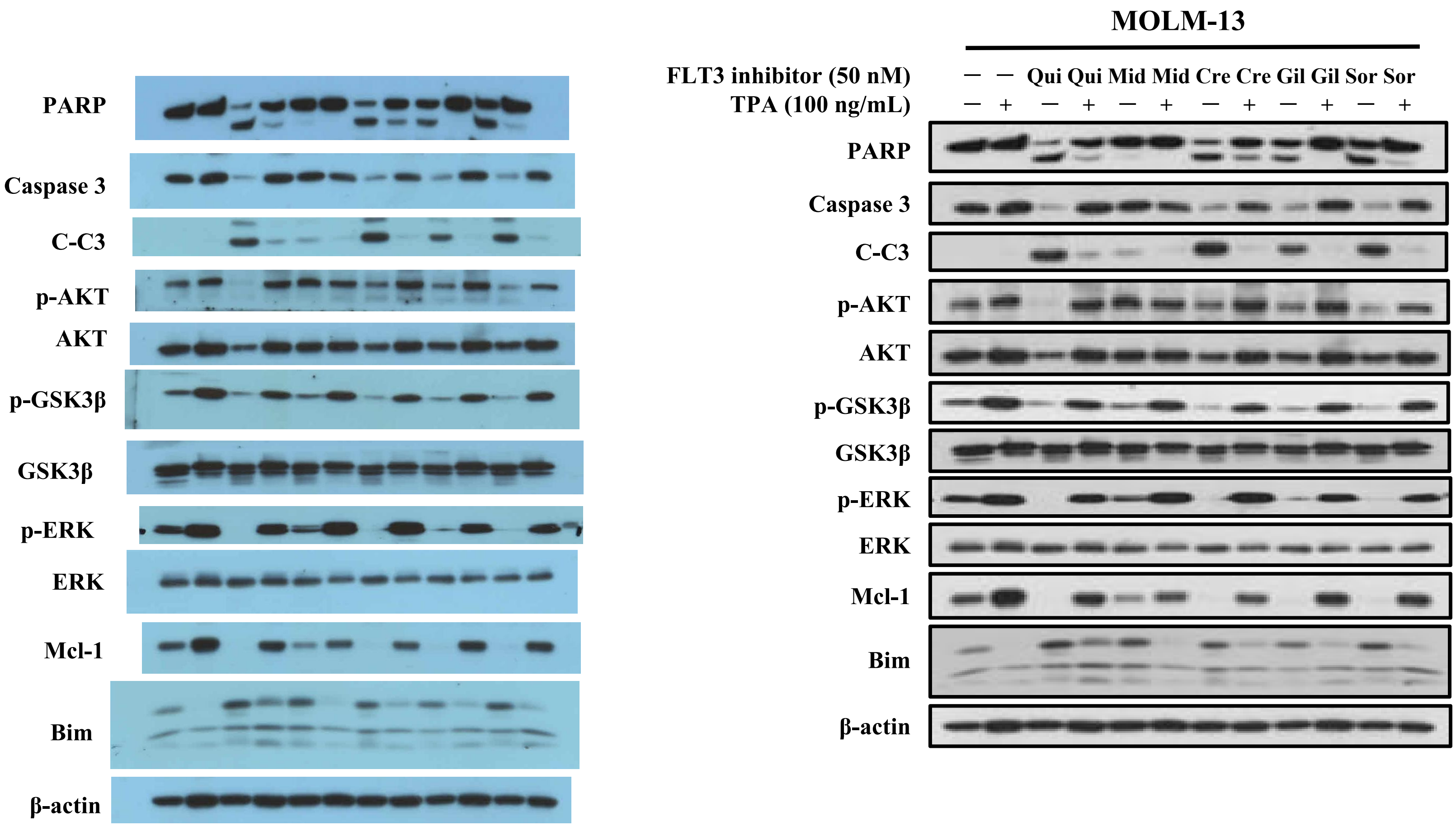
MOLM-13
FLT3 inhibitor (50 nM) — — Qui Qui Mid Mid Cre Cre Gil Gil Sor Sor
TPA (100 ng/mL) — + — + — + — + — + — +
PARP
Caspase 3
C-C3
p-AKT
AKT
p-GSK3β
GSK3β
p-ERK
ERK
Mcl-1
Bim
β-actin

Fig.2D

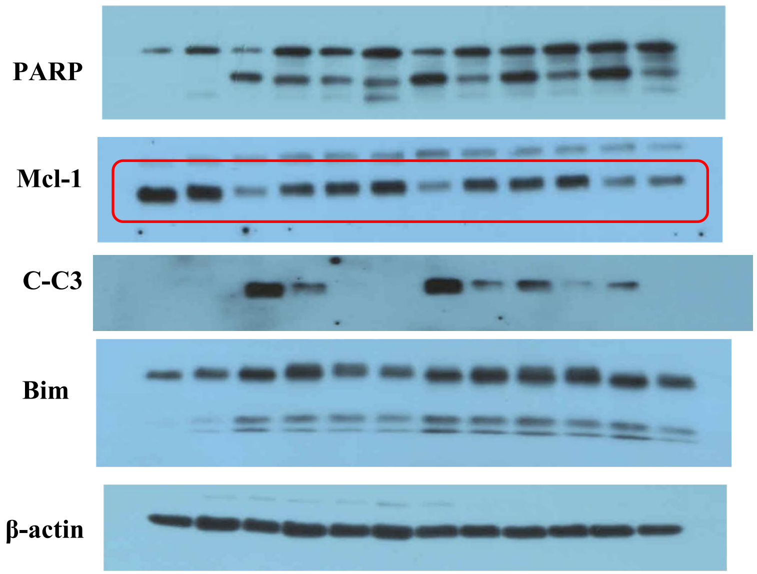
PARP
Mcl-1
C-C3
Bim
β-actin

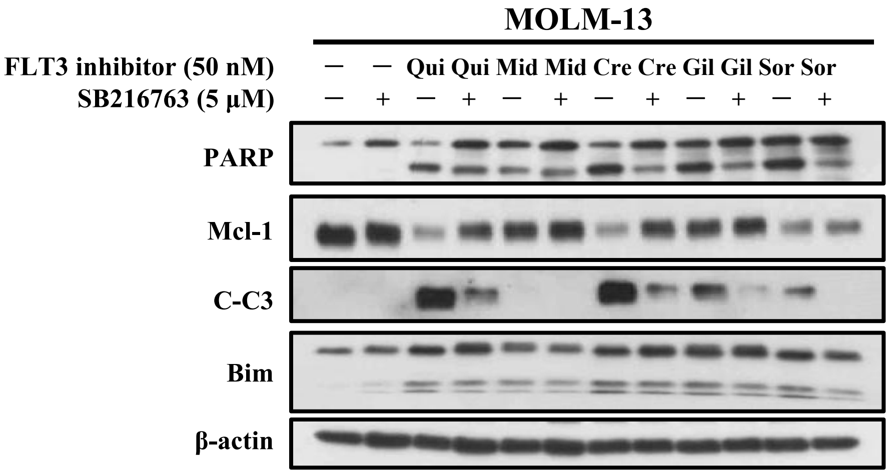
MOLM-13
FLT3 inhibitor (50 nM) — — Qui Qui Mid Mid Cre Cre Gil Gil Sor Sor
SB216763 (5 µM) — + — + — + — + — + — +
PARP
Mcl-1
C-C3
Bim
β-actin

Fig.2F

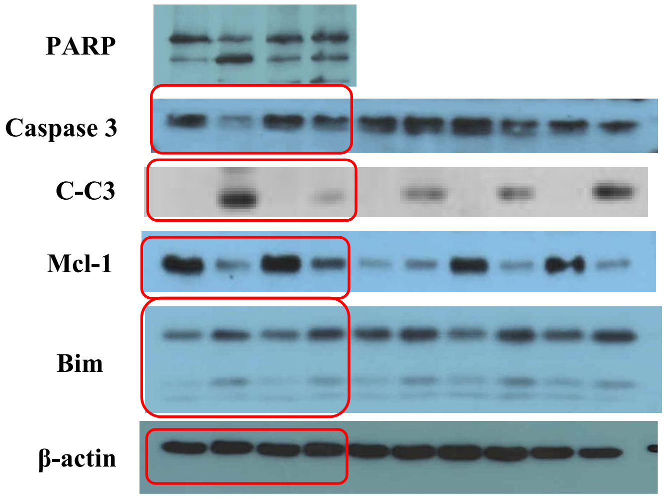
PARP
Caspase 3
C-C3
Mcl-1
Bim
β-actin

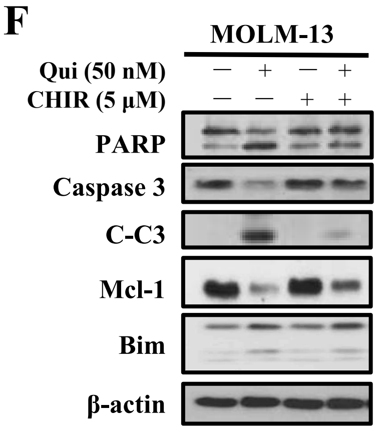
F
MOLM-13
Qui (50 nM) — + — +
CHIR (5 μM) — — + +
PARP
Caspase 3
C-C3
Mcl-1
Bim
β-actin

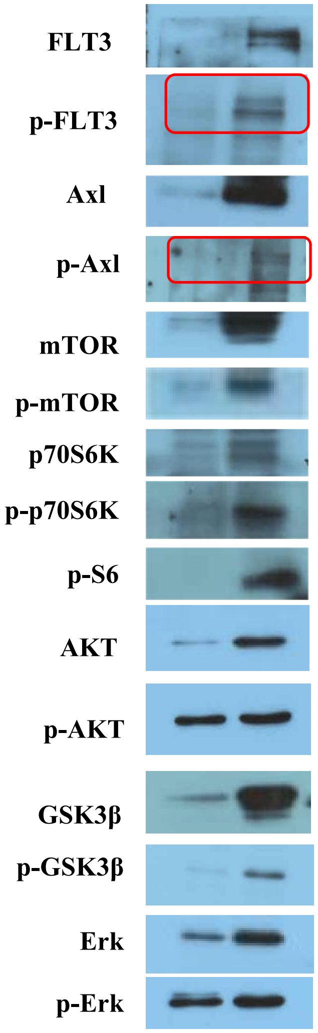
FLT3
p-FLT3
Axl
p-Axl
mTOR
p-mTOR
p70S6K
p-p70S6K
p-S6
AKT
p-AKT
GSK3β
p-GSK3β
Erk
p-Erk

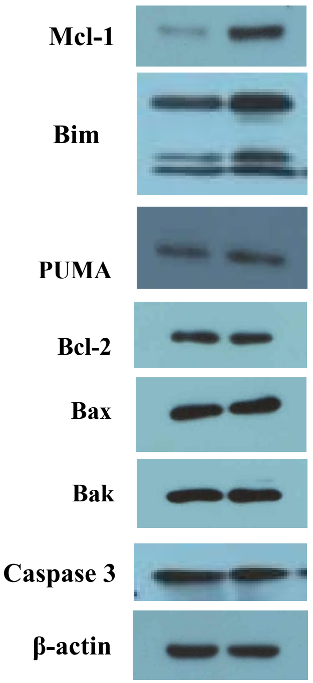
Mcl-1
Bim
PUMA
Bcl-2
Bax
Bak
Caspase 3
β-actin

Fig. 3F

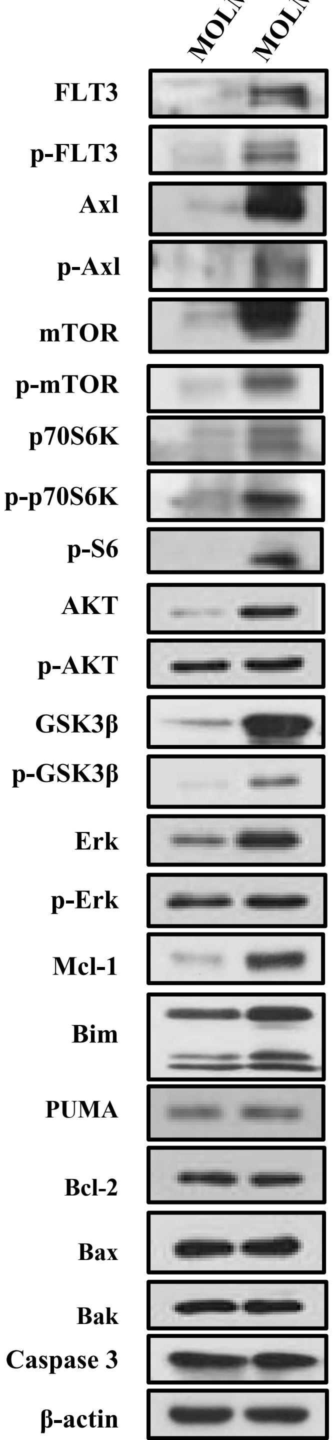
FLT3
p-FLT3
Axl
p-Axl
mTOR
p-mTOR
p70S6K
p-p70S6K
p-S6
AKT
p-AKT
GSK3β
p-GSK3β
Erk
p-Erk
Mcl-1
Bim
PUMA
Bcl-2
Bax
Bak
Caspase 3
β-actin

Fig.4C

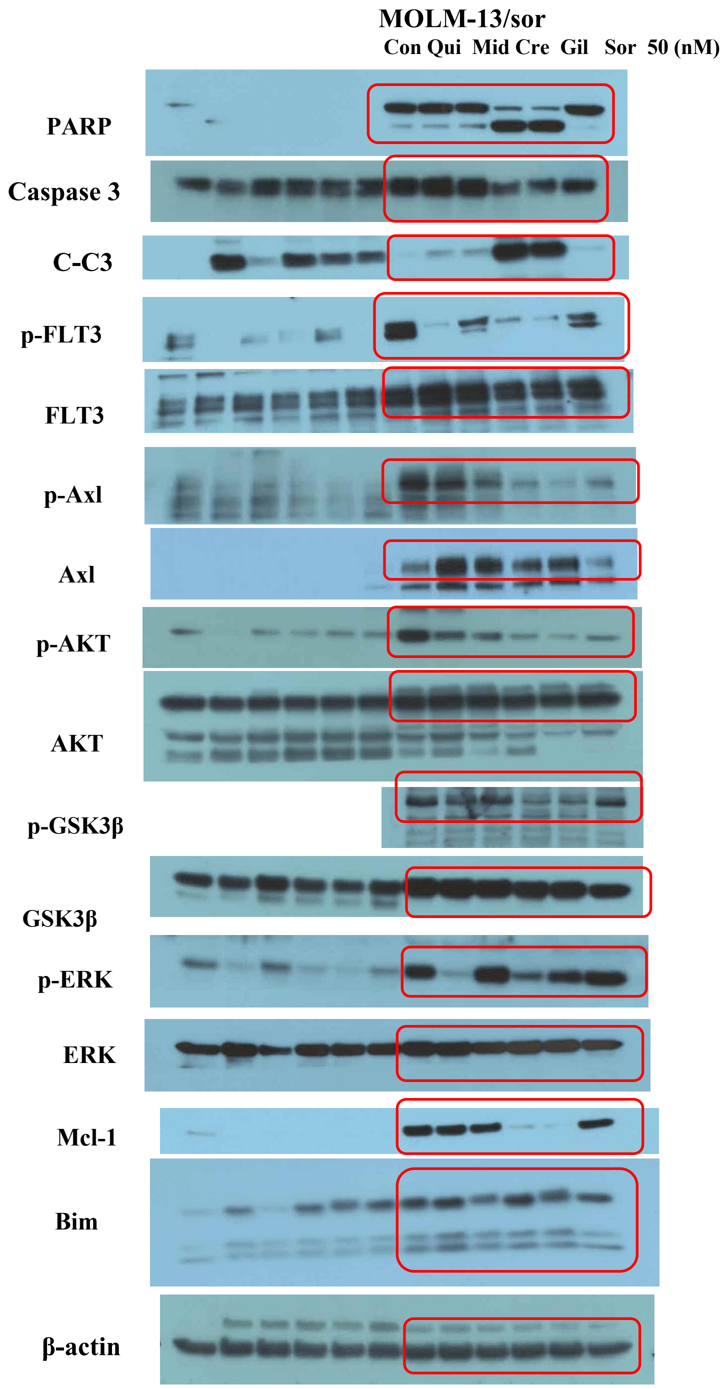
MOLM-13/sor
Con Qui Mid Cre Gil Sor 50 (nM)
PARP
Caspase 3
C-C3
p-FLT3
FLT3
p-Axl
Axl
p-AKT
AKT
p-GSK3β
GSK3β
p-ERK
ERK
Mcl-1
Bim
β-actin

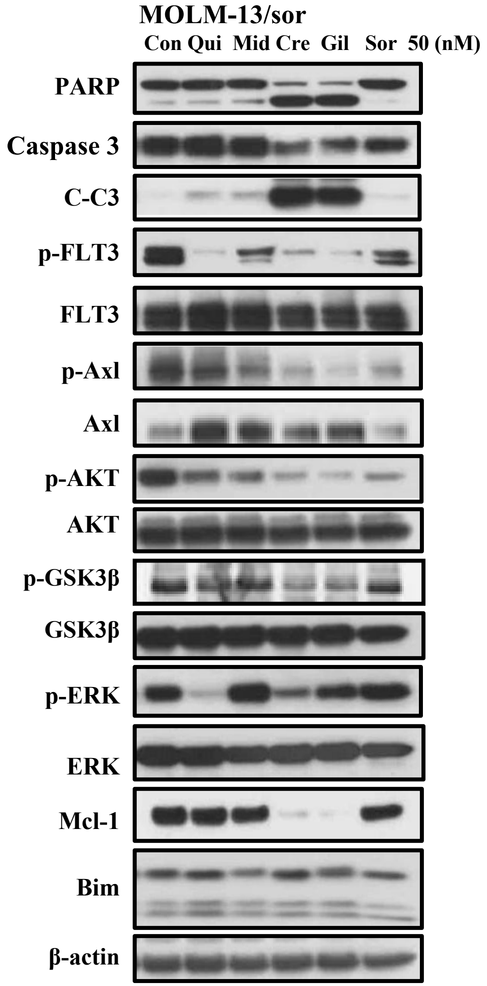
MOLM-13/sor
Con Qui Mid Cre Gil Sor 50 (nM)
PARP
Caspase 3
C-C3
p-FLT3
FLT3
p-Axl
Axl
p-AKT
AKT
p-GSK3β
GSK3β
p-ERK
ERK
Mcl-1
Bim
β-actin

Fig. 4E

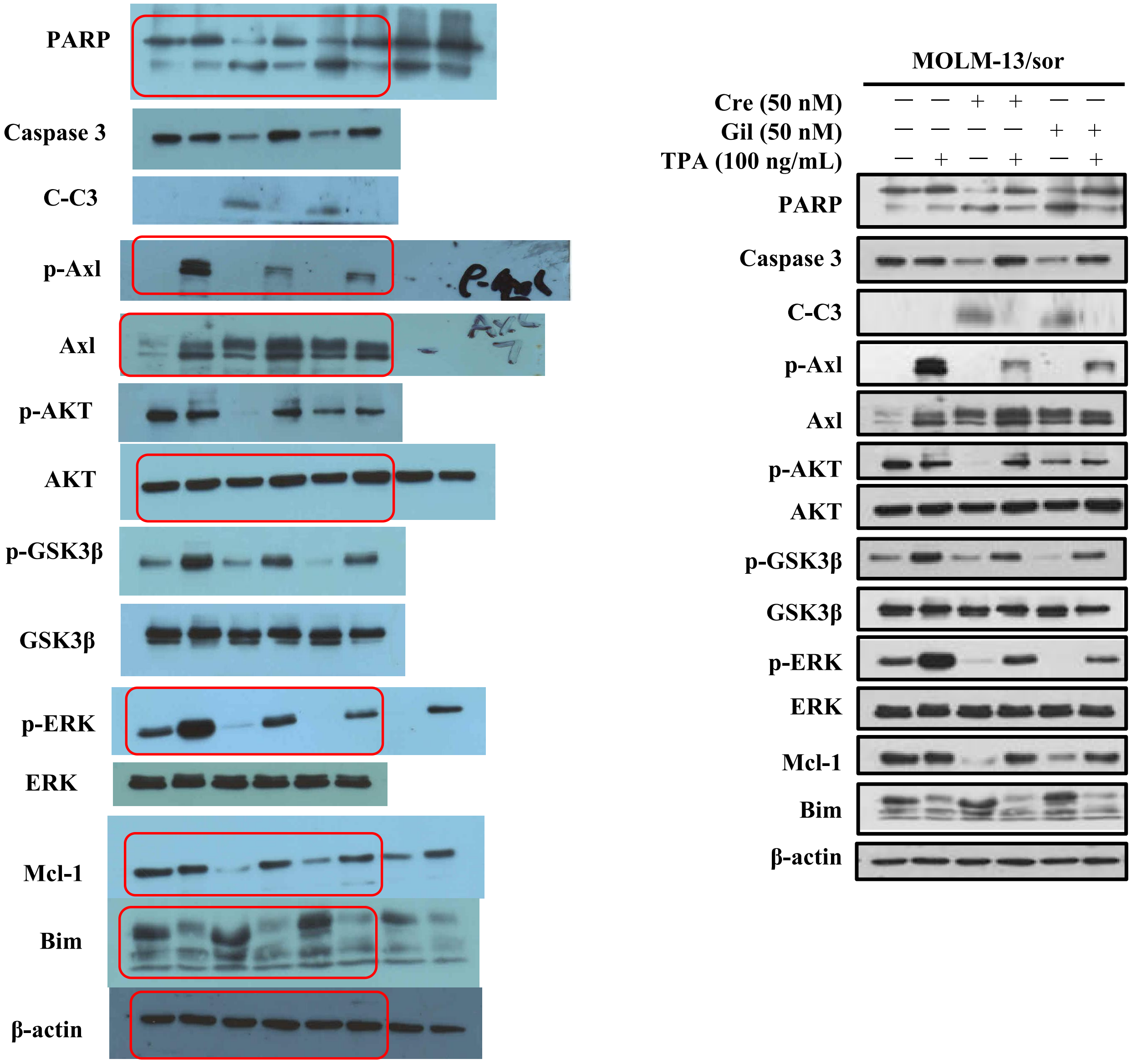
PARP
Caspase 3
C-C3
p-Axl
Axl
p-AKT
AKT
p-GSK3β
GSK3β
p-ERK
ERK
Mcl-1
Bim
β-actin
MOLM-13/sor
Cre (50 nM) — — + + — —
Gil (50 nM) — — — — + +
TPA (100 ng/mL) — + — + — +
PARP
Caspase 3
C-C3
p-Axl
Axl
p-AKT
AKT
p-GSK3β
GSK3β
p-ERK
ERK
Mcl-1
Bim
β-actin

Fig. 4G

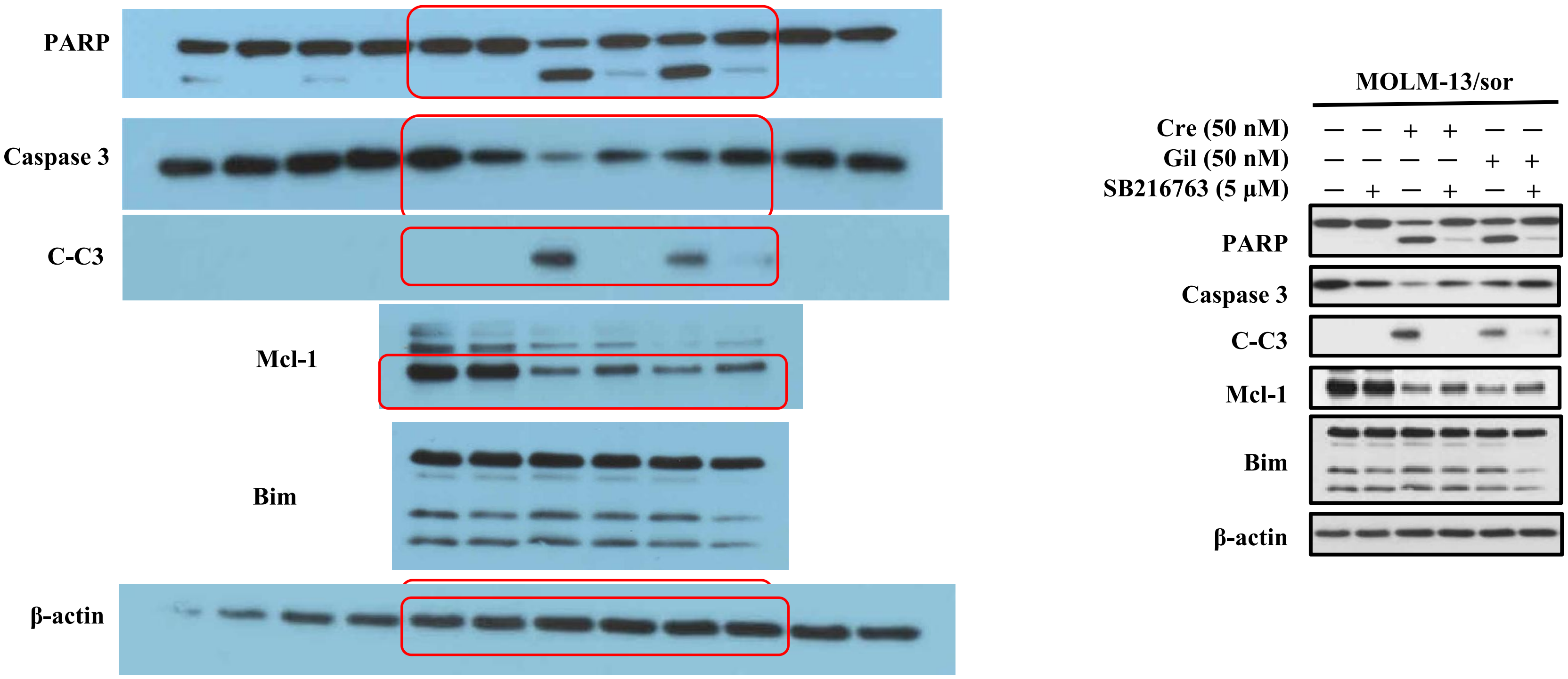
PARP
Caspase 3
C-C3
Mcl-1
Bim
β-actin
MOLM-13/sor
Cre (50 nM)
Gil (50 nM)
SB216763 (5 μM)
PARP
Caspase 3
C-C3
Mcl-1
Bim
β-actin

Fig. 5B

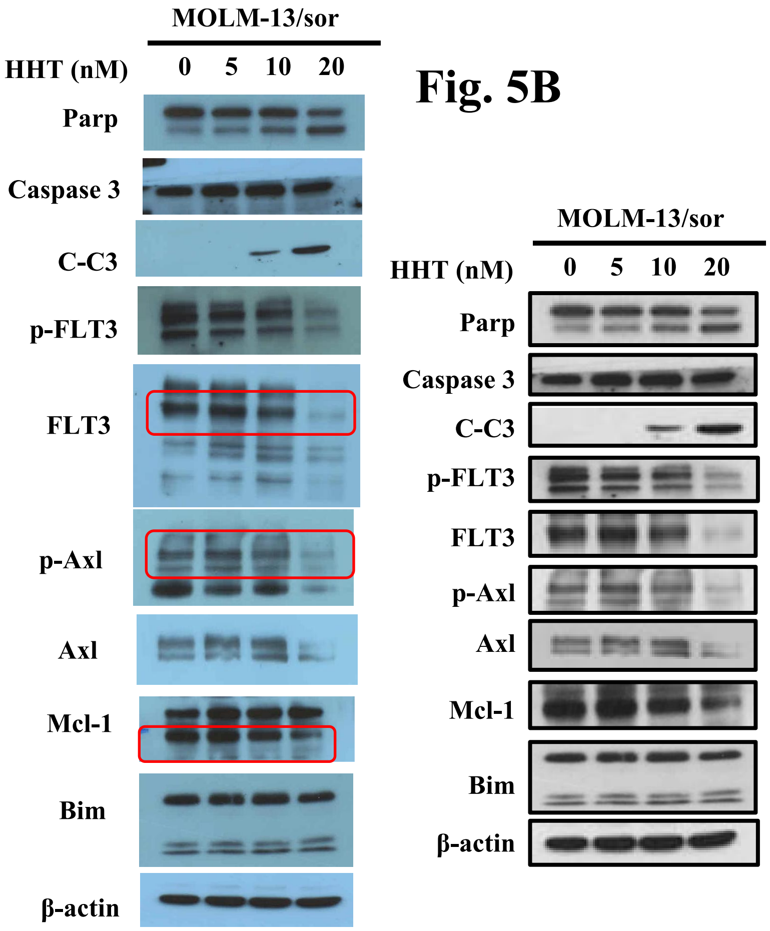
MOLM-13/sor
HHT (nM)
0
5
10
20
Parp
Caspase 3
C-C3
p-FLT3
FLT3
p-Axl
Axl
Mcl-1
Bim
β-actin

Fig. 5D

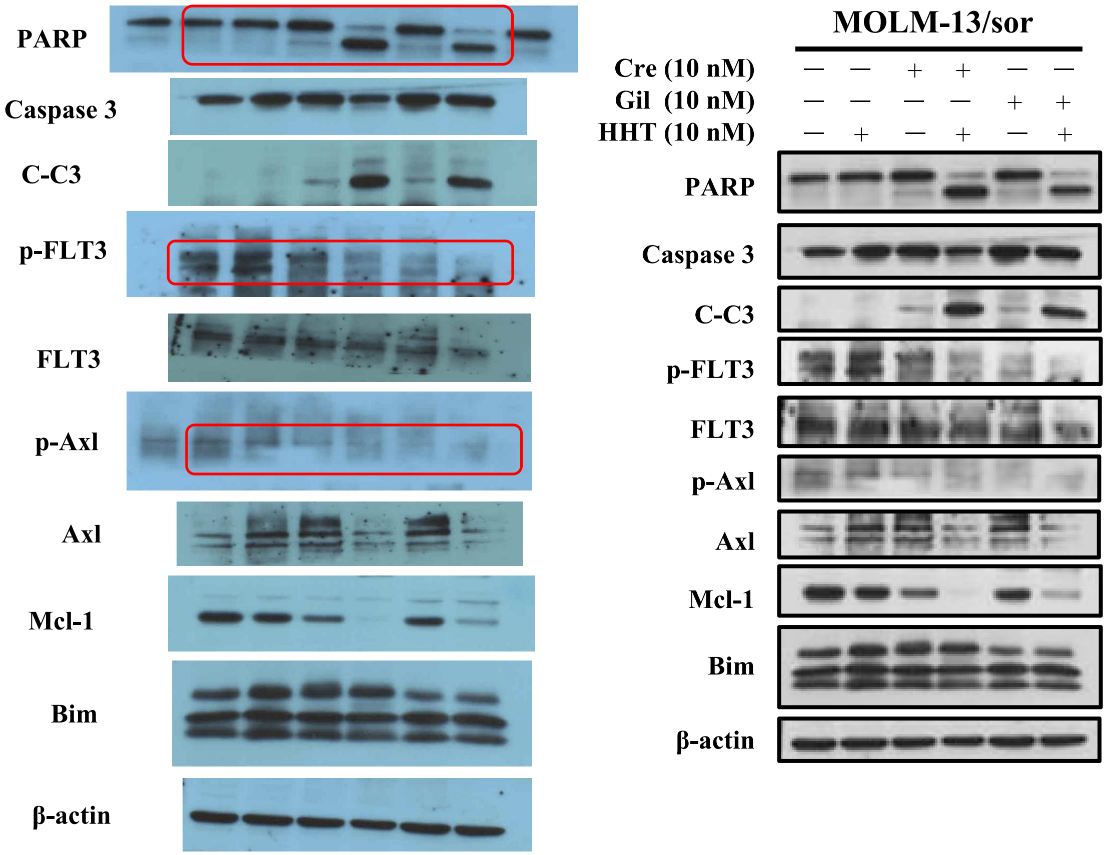
PARP
Caspase 3
C-C3
p-FLT3
FLT3
p-Axl
Axl
Mcl-1
Bim
β-actin
MOLM-13/sor
Cre (10 nM)
Gil (10 nM)
HHT (10 nM)

Fig. 6C

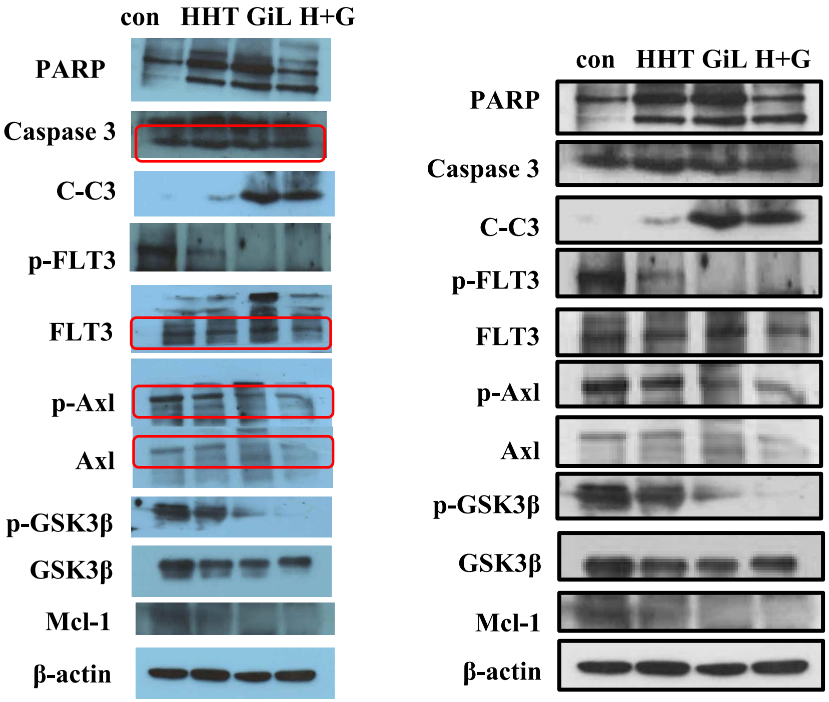
con HHT GiL H+G
PARP
Caspase 3
C-C3
p-FLT3
FLT3
p-Axl
Axl
p-GSK3β
GSK3β
Mcl-1
β-actin
con HHT GiL H+G
PARP
Caspase 3
C-C3
p-FLT3
FLT3
p-Axl
Axl
p-GSK3β
GSK3β
Mcl-1
β-actin

Fig. S1B

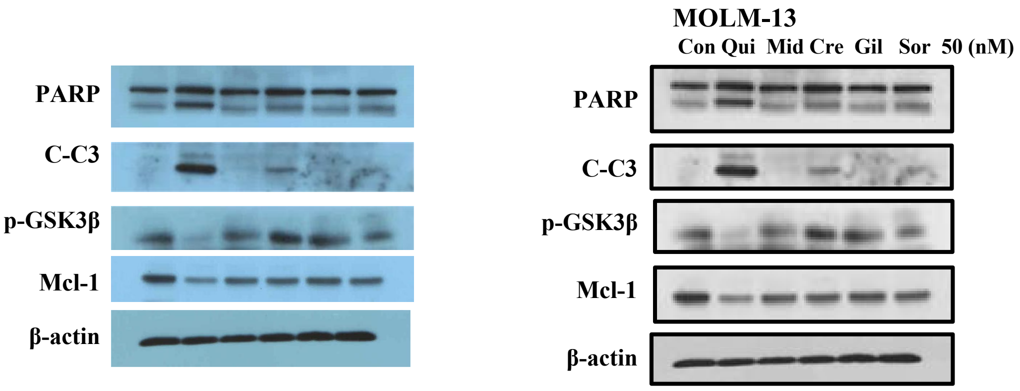

MOLM-13
Con Qui Mid Cre Gil Sor 50 (nM)
PARP
C-C3
p-GSK3β
Mcl-1
β-actin
PARP
C-C3
p-GSK3β
Mcl-1
β-actin

Fig. S2B

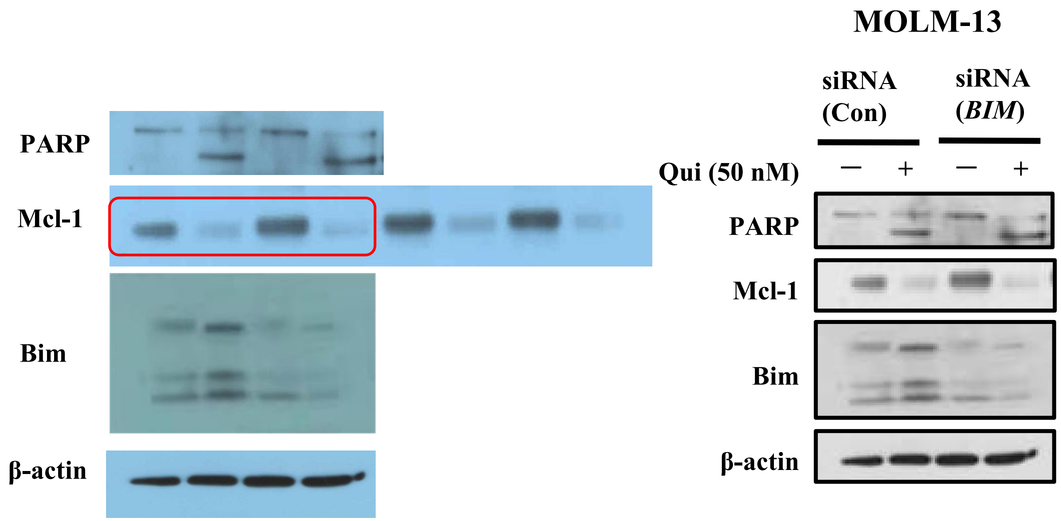

Fig. S3A

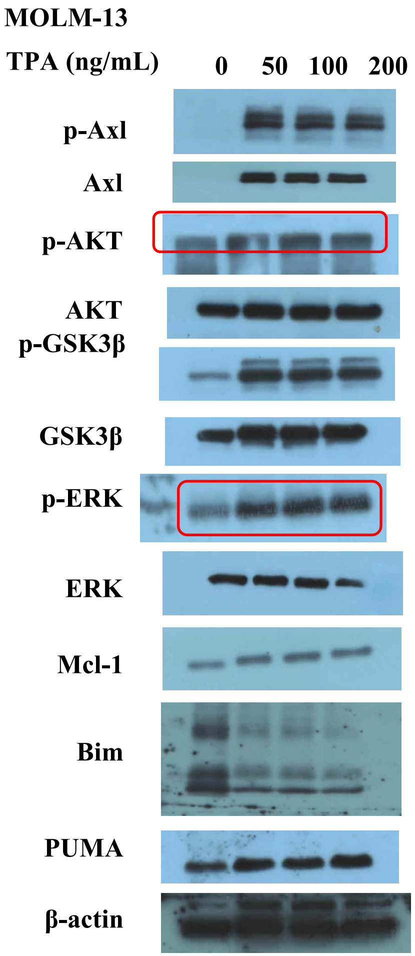

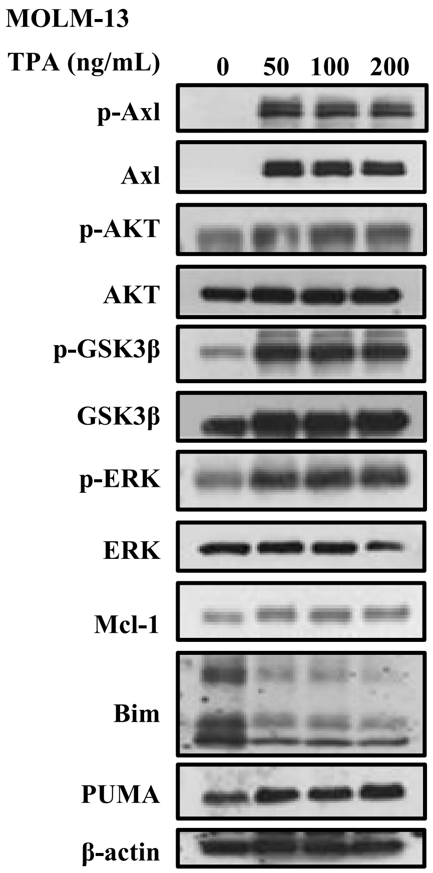

Fig. S3B

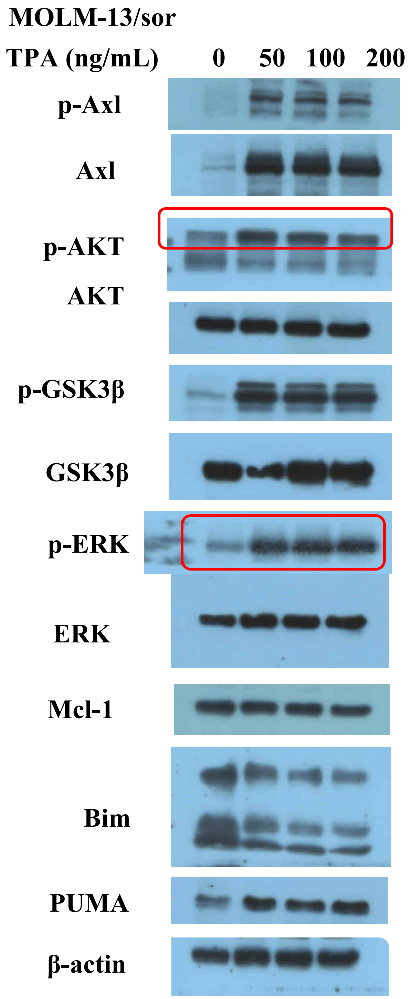

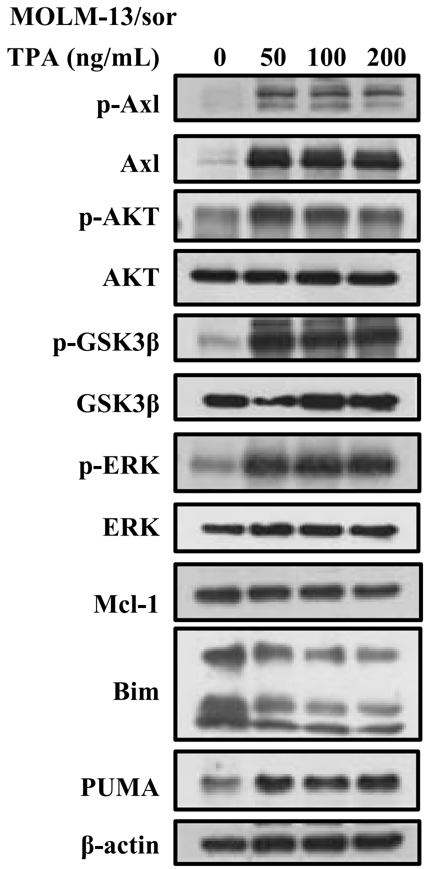

Fig.S4B

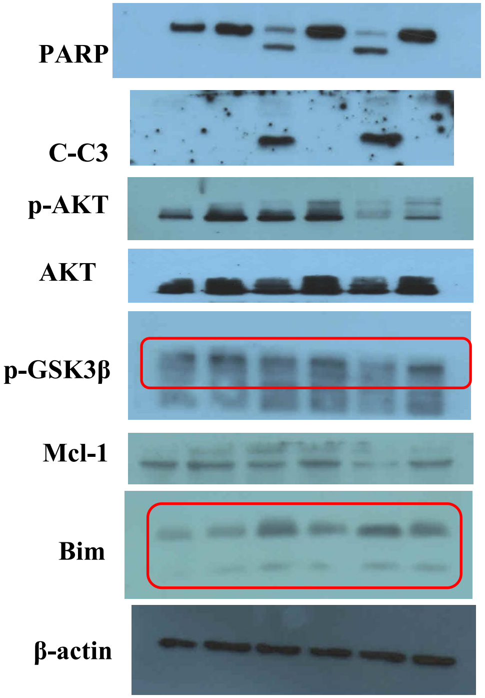
PARP
C-C3
p-AKT
AKT
p-GSK3β
Mcl-1
Bim
β-actin

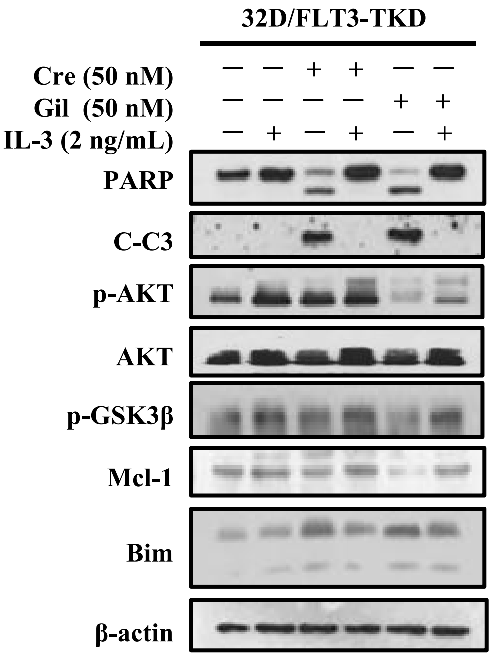
32D/FLT3-TKD
Cre (50 nM) — — + + — —
Gil (50 nM) — — — — + +
IL-3 (2 ng/mL) — + — + — +
PARP
C-C3
p-AKT
AKT
p-GSK3β
Mcl-1
Bim
β-actin

Fig. S5B

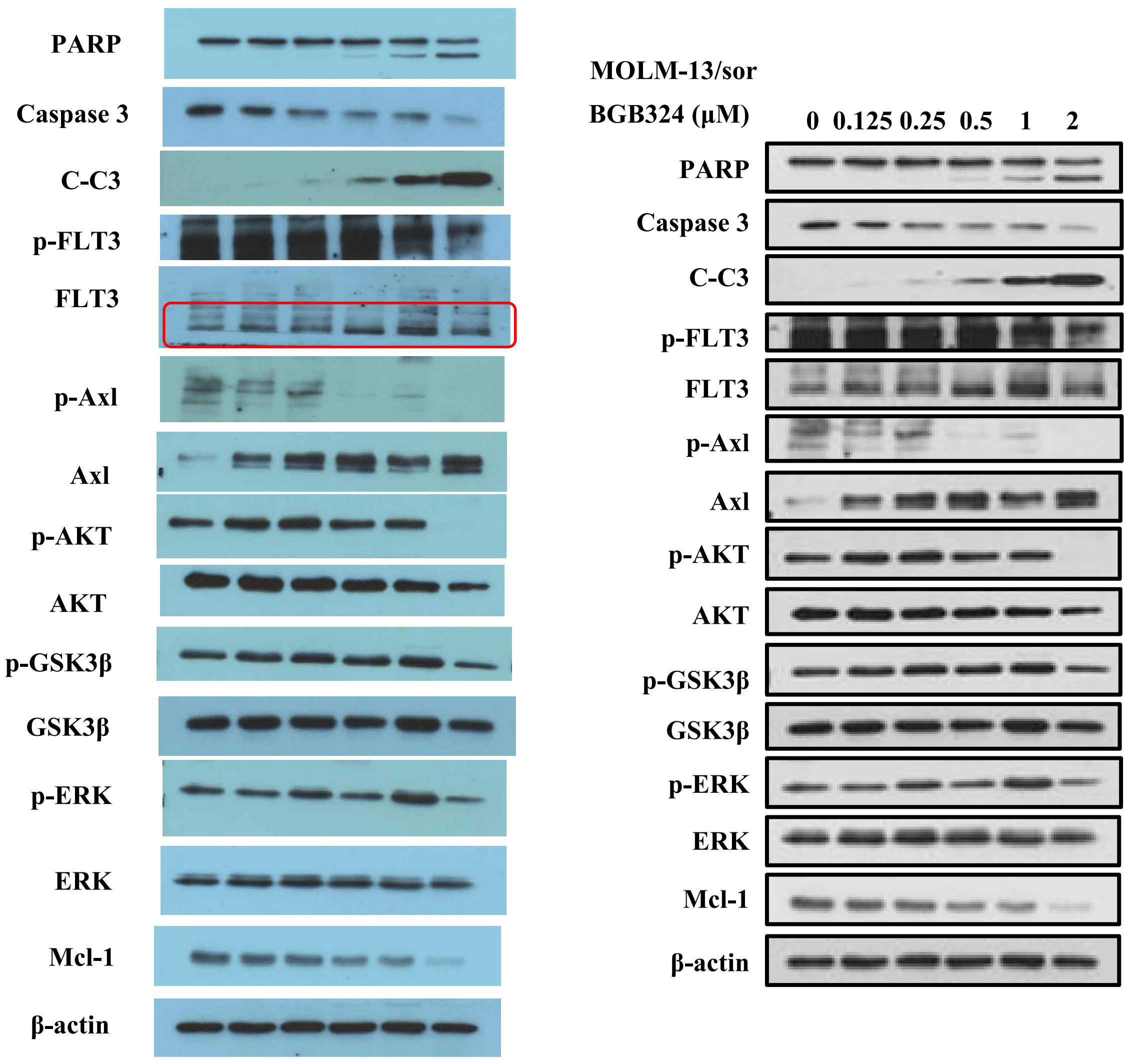
PARP
Caspase 3
C-C3
p-FLT3
FLT3
p-Axl
Axl
p-AKT
AKT
p-GSK3β
GSK3β
p-ERK
ERK
Mcl-1
β-actin
MOLM-13/sor
BGB324 (μM)
0 0.125 0.25 0.5 1 2
PARP
Caspase 3
C-C3
p-FLT3
FLT3
p-Axl
Axl
p-AKT
AKT
p-GSK3β
GSK3β
p-ERK
ERK
Mcl-1
β-actin

Fig. S5D

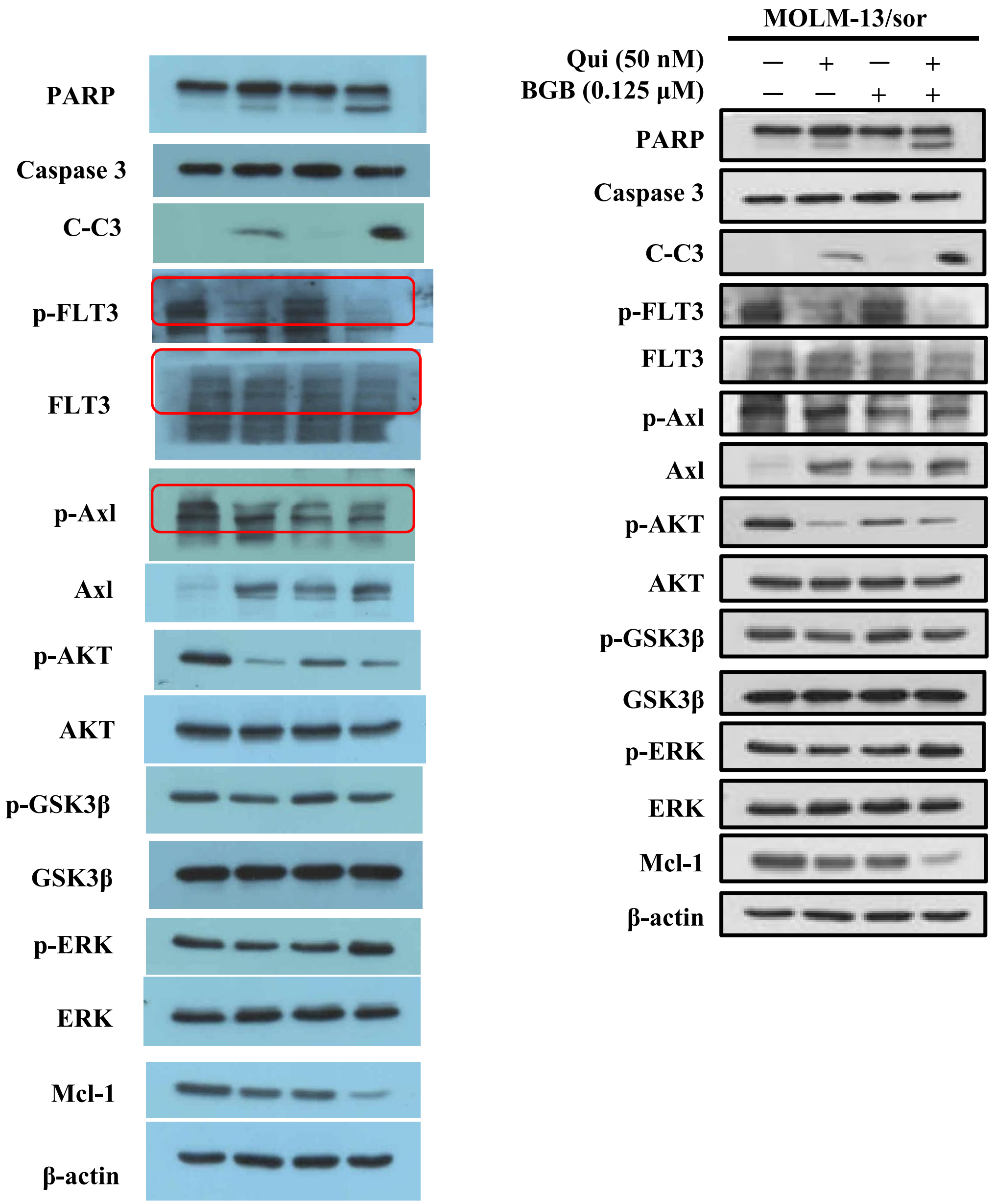

MOLM-13/sor
Qui (50 nM) — + — +
BGB (0.125 μM) — — + +
PARP
Caspase 3
C-C3
p-FLT3
FLT3
p-Axl
Axl
p-AKT
AKT
p-GSK3β
GSK3β
p-ERK
ERK
Mcl-1
β-actin

Fig. S5E

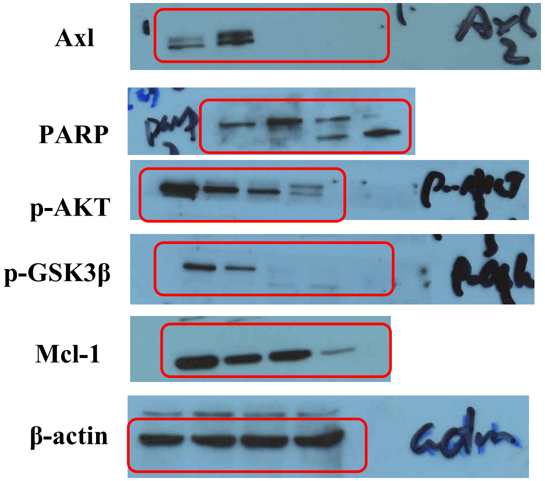
Axl
PARP
p-AKT
p-GSK3β
Mcl-1
β-actin

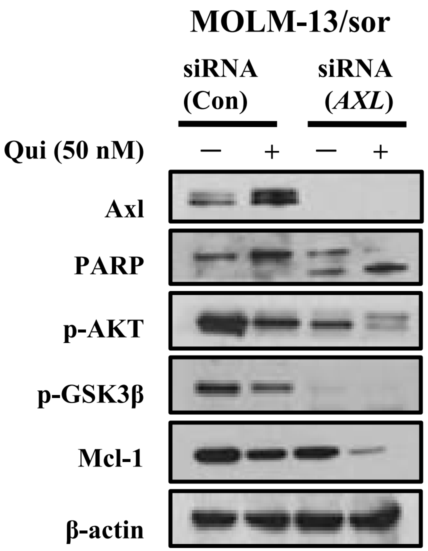
MOLM-13/sor
siRNA (Con)
siRNA (*AXL*)
Qui (50 nM)
– + – +
Axl
PARP
p-AKT
p-GSK3β
Mcl-1
β-actin

Fig. S6A

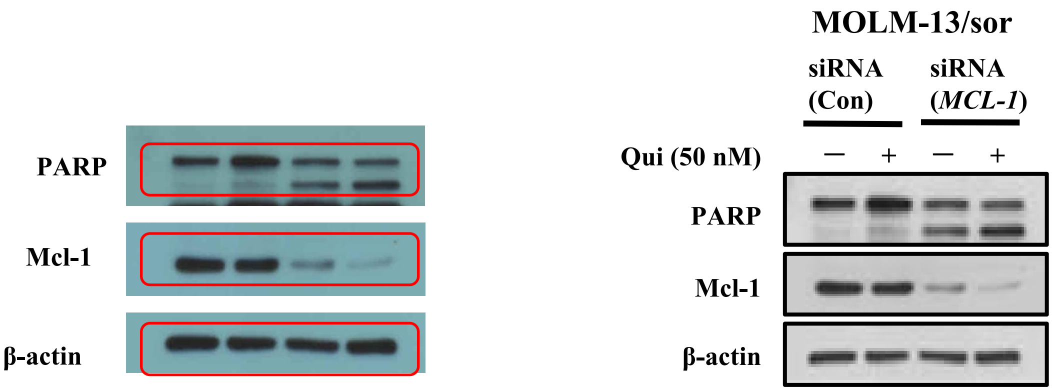

Fig. S6B

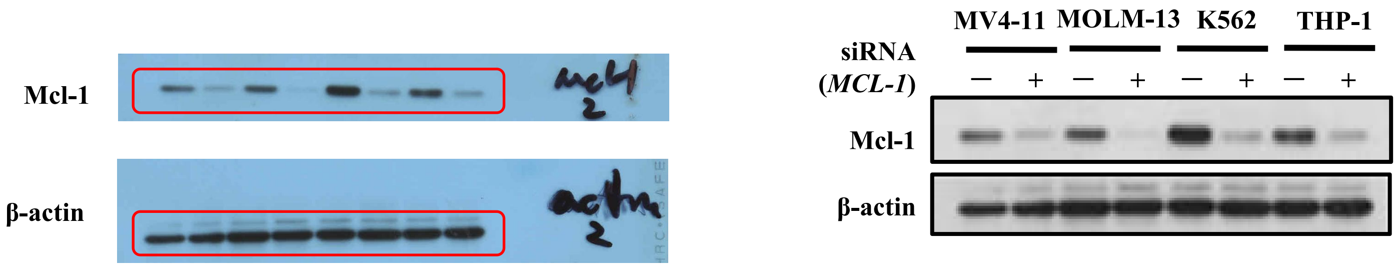

Supplement: Supplementary file 3 — Original Data File [file 41420_2023_1317_MOESM3_ESM.pdf]
